# Systematic cross-species comparison of prefrontal cortex functional networks targeted via transcranial magnetic stimulation

**DOI:** 10.1162/imag_a_00243

**Published:** 2024-07-24

**Authors:** Taylor Berger, Ting Xu, Alexander Opitz

**Affiliations:** Department of Biomedical Engineering, University of Minnesota, Minneapolis, MN, United States; Child Mind Institute, New York, NY, United States

**Keywords:** transcranial magnetic stimulation, non-human primates, finite element analysis, functional networks

## Abstract

Transcranial magnetic stimulation (TMS) is a non-invasive brain stimulation method that safely modulates neural activity in vivo. Its precision in targeting specific brain networks makes TMS invaluable in diverse clinical applications. For example, TMS is used to treat depression by targeting prefrontal brain networks and their connection to other brain regions. Despite its widespread use, however, the underlying neural mechanisms of TMS are not completely understood. Non-human primates (NHPs) offer an ideal model to study TMS mechanisms through invasive electrophysiological recordings. As such, bridging the gap between NHP experiments and human applications is imperative to ensure translational relevance. Here, we systematically compare the TMS-targeted functional networks in the prefrontal cortex in humans and NHPs. We combine TMS electric field modeling in humans and macaques with resting-state functional magnetic resonance imaging (fMRI) data to compare the functional networks targeted via TMS across species. Distinct stimulation zones in macaque and human models arose, each exhibiting differences in impacted networks (macaque: Frontoparietal Network, Somatomotor Network; human: Frontoparietal Network, Default Network). We identified differences in brain gyrification and functional organization across species as the underlying cause of found network differences. The TMS-network profiles we identified will allow researchers to establish consistency in network stimulation across species, aiding in the translational efforts to develop improved TMS functional network targeting approaches.

## 1 Introduction

Transcranial magnetic stimulation (TMS) is a non-invasive brain stimulation method that can safely modulate neural activity in vivo (Rossi et al., 2009). This technique applies a rapidly changing magnetic field through a coil placed at the scalp which induces an electric field in underlying brain regions. TMS has high spatial resolution (Deng et al., 2013;Opitz et al., 2011), which enables targeting of specific brain networks. As such, TMS is an emerging therapeutic option for several neurological and psychiatric disorders (Lefaucheur et al., 2014). TMS is approved by the Food and Drug Administration to treat depression, obsessive-compulsive disorders, and smoking cessation, with ongoing clinical trials for other indications. Despite its increasing applications in clinical and basic research, the physiological outcomes of TMS are known to be variable across individuals (Hamada et al., 2013;López-Alonso et al., 2014). One potential reason is the imprecise targeting of brain circuits with TMS. Due to differences in neuroanatomy, the induced electric fields can differ significantly across individuals. Computational models based on the finite element method (FEM) are used to estimate the TMS-induced electric field in individually realistic head models (Windhoff et al., 2013). This modeling technique allows researchers to predict the impact of unique anatomical patterns, such as brain gyrification and cerebrospinal fluid (CSF) thickness, on stimulation (Opitz et al., 2011;Thielscher et al., 2011). It is important to acknowledge that anatomy and function are not always one to one. FEM modeling, while adept at capturing unique anatomical features, does not incorporate aspects of functional brain organization. Understanding the functional organization is particularly important for targeting higher-order association areas that are implicated in the treatment of depression (Rizvi & Khan, 2019;Schutter, 2009;Zhao et al., 2022).

In higher-order association areas, the relationship between brain anatomical landmarks and functional behavior remains subject to ongoing research (Amiez et al., 2006;Goulas et al., 2012;Margulies & Petrides, 2013). Resting-state functional magnetic resonance imaging (r-fMRI) is a popular method to map functional brain networks in vivo (Power et al., 2011;Smith et al., 2013). r-fMRI enables precision mapping of individual functional networks with high test-retest reliability (Gratton et al., 2020;Laumann et al., 2015). Consequently, researchers have suggested using r-fMRI to guide TMS targeting (Fox et al., 2012;Oathes et al., 2021). Network-guided TMS has the potential to account for individualized functional networks and target symptom-specific brain networks (Siddiqi et al., 2020). Our research team has further developed this method and combined it with FEM modeling (Opitz et al., 2016), identifying distinct r-fMRI networks stimulated by TMS-induced electric fields. This method allows researchers to integrate both anatomical and functional aspects of TMS targeting. While these methods have provided important tools to predict TMS stimulation of functional networks, they need further experimental validation. In humans, mostly indirect measurements of TMS physiological effects are available which has hampered efforts to elucidate TMS network mechanisms.

Research in non-human primates (NHPs) has created opportunities to explore these assumptions and study TMS mechanisms during invasive physiological recordings. NHPs are ideally suited to investigate TMS neural mechanisms (de Lima-Pardini et al., 2023;Hanlon et al., 2021) because they have human-like cortical complexity and prefrontal cortex development (Lear et al., 2022). This allows researchers to conduct translational studies within prefrontal brain regions, a common target area in treating depression. Invasive recordings in NHPs have allowed researchers to study the neural effects of TMS with precision not available in humans (Mueller et al., 2014;Perera et al., 2023;Romero et al., 2019). Despite its unique opportunities, the translation between NHP and human TMS applications is not straightforward. The anatomical features (i.e., size, gyrification) (Alekseichuk et al., 2019) and functional organization (Xu et al., 2020) lack one-to-one homology to human models. Notably, the evolutionary expansion of the human neocortex results in a highly convoluted cortex, especially in higher-order regions (Donahue et al., 2018;Mars et al., 2018;Van Essen, 2004;Van Essen & Dierker, 2007). Salience, frontoparietal (FPN), default networks (DN), and their interactions exhibit greater variations in humans than those observed in NHPs (Ardesch et al., 2019;Donahue et al., 2018;van den Heuvel et al., 2023). This disparity highlights the need for a bidirectional translational pipeline that can map TMS functional network targeting between humans and NHPs.

Here, we develop an integrated cross-species framework that combines interspecies anatomical alignment with r-fMRI to map TMS functional networks between humans and NHPs. This is based on our recently developed cross-species functional alignment method (Xu et al., 2020) that enables a quantitative comparison of functional homology across humans and NHPs. We focus on the prefrontal cortex due to its intricate complexity across species and crucial role in TMS clinical applications. We systematically compare the functional networks targeted with TMS in the prefrontal cortex between macaques and humans to highlight commonalities and differences in functional networks across species and investigate factors leading to these observed differences.

## 2 Methods

### 2.1 Overview

**Fig. 1.**
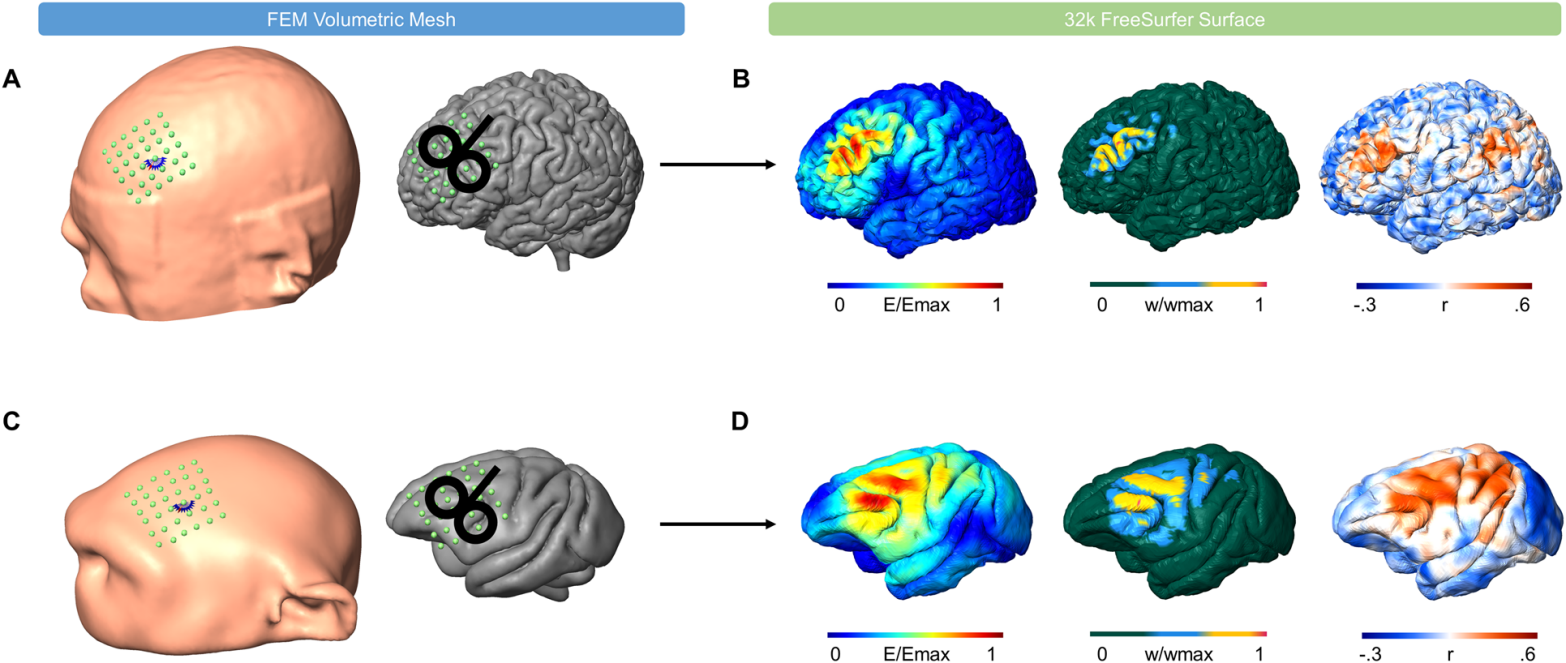
Overview of the integrated TMS-rfMRI network modeling pipeline. The TMS-rfMRI network modeling pipeline compares the ability of TMS to target established functional connectivity networks across species. Individual volumetric head models were used to simulate TMS electric fields over a stimulation grid (6 x 6 coil locations, 12 coil orientations 0°-165°) (A) centered over F3 on humans and (C) the left prefrontal cortex on macaques. (B, D) The resulting TMS electric fields (left) were interpolated to a common space (32k FreeSurferLR surface). Seed regions (middle) were correlated with individual r-fMRI data to calculate TMS-functional connectivity profiles (right).

We developed a cross-species integrated TMS resting-state functional Magnetic Resonance Imaging (rfMRI) network modeling pipeline to compare TMS-targeted functional networks in humans and non-human primates (Fig. 1). This pipeline simulates TMS electric fields using individual FEM head models (Fig. 1A, C) given specified coil configurations. The resulting TMS electric fields are used as a seed region for individual rfMRI analysis (Fig. 1B, D) to extract TMS-targeted networks based on Yeo functional networks. Finally, we compared the targeted functional networks across species by utilizing the previously established macaque-human cortical transforms (Xu et al., 2020). In this analysis, we focus on the left prefrontal cortex. This brain region is highly relevant for therapeutic applications of TMS (van den Heuvel et al., 2023;Wassermann & Lisanby, 2001). Further, the prefrontal cortex is a higher-order multimodal association area, which is richly connected to several other brain regions (Friedman & Robbins, 2022). Thus, investigating which functional networks are targeted by TMS in this region is of high relevance for research and clinical applications.

### 2.2 Ethics statement

This work utilizes pre-existing data from third-party sources. No additional ethical approval was required.

### 2.3 Human dataset

We randomly selected MRI data from 10 unrelated, healthy young adults (age: 22–35, 5 females and 5 males) from the Human Connectome Project (HCP) s1200 release (Van Essen et al., 2012). Images included T1-weighted MP-RAGE, T2-weighted MP-SPACE MRI, and an r-fMRI scan. The r-fMRI scan used within this analysis was acquired on day 1 of the HCP S1200 fMRI protocol (TR = 0.72 s, 2 mm isotropic) and contained a single 15-minute run (phase encoding left-right) for each participant. The Minimal Preprocessing Pipeline (MPP) (Glasser et al., 2013) from the HCP s1200 release (WU_Minn, 2017) was applied to all structural imaging files in the HCP preprocessing pipeline. Briefly, MPP includes spatial/artifact distortion removal, cross-modal registration, surface generation, and alignment to a symmetric fsLR-32k template surface (Van Essen et al., 2012). The functional MPP includes motion correction, distortion correction, ICA-FIX denoising, and spatial smoothness (FWHM = 2 mm) (Smith et al., 2013). We used the 32k FreeSurfer LR (fsLR) aligned surfaces in native space for each participant to capture the individual morphology of the brain shape. The aligned 32k fsLR surfaces contain 32,492 vertices per hemisphere that are comparable across participants.

### 2.4 Macaque dataset

Rhesus macaque (*Macaca mulatta*) MRI data are publicly available from the Oxford (anesthetized) dataset within the open-source NHP consortium, PRIMatE-Data Exchange (PRIME-DE) (Milham et al., 2018). The r-fMRI scan consisted of a single 53.3-minute run per animal under anesthesia with isoflurane. The ethics approval for all animal care and experiments was carried out in compliance with the UK Animals (Scientific Procedures) Act of 1986. The details of the animal housing, anesthesia protocols, and MRI acquisition were reported in previous studies (Noonan et al., 2014) and PRIME-DE (Milham et al., 2018). The macaque imaging data underwent preprocessing using a customized HCP-like pipeline (Xu et al., 2015,^2018^,^2019^). Briefly, the preprocessing of r-fMRI includes slice timing, motion correction, co-registration, nuisance regression (Friston’s 24 motion parameters, mean signal of white matter (WM) and CSF), and band-pass filtering (0.01–0.1 Hz). Finally, the preprocessed data were projected from the volume to the surface space and smoothed (FWHM = 2 mm) along the surface. To ensure the accuracy of the individual FEM model, quality assessment and visual inspection were conducted on the co-registration and surface reconstruction steps. Ten male NHPs (age = 4.3 years ± 0.4 years, weight = 7.3 kgs ± 1.4 kgs) were included in our final analysis (Milham et al., 2018). Like HCP’s MPP, 32k FreeSurfer surfaces were generated per hemisphere for each animal to enable direct comparison across animals within the macaque dataset.

### 2.5 Estimation of TMS induced electric fields in humans

We created individual FEM models for each of the 10 participants based on the high-resolution T1- and T2-weighted images using SimNIBS 3.2 (Thielscher et al., 2015). We constructed a stimulation grid of 36 coil locations centered on F3 (6 x 6, left-right and anterior-posterior directions, 10 mm spacing) on each FEM head model, as illustrated inFigure 1A. We designed the grid to cover different TMS targeting strategies for the left dorsolateral prefrontal cortex (DLPFC) (Avery et al., 2006;George et al., 1995,^2010^;Herbsman et al., 2009;Herwig et al., 2001;Pascual-Leone et al., 1996). At each grid point, we simulated 12 distinct coil orientations, stepping in 15-degree increments, covering a 180-degree half-circle (0-degrees corresponding to the coil handle position along the midline). For each participant, we simulated 432 TMS electric fields (36 coil locations x 12 coil orientations) using a Figure-8 Magstim coil with a 70 mm diameter. We interpolated the calculated electric field strength from the FEM volumetric mesh to the pial surface of the 32k fsLR subject surface using an iterative closest point (ICP) algorithm (Rusinkiewicz & Levoy, 2001).Figure 1B(left) depicts an example of a simulated electric field distribution.

### 2.6 Estimation of TMS-induced electric fields in macaques

To create a realistic FEM head model, we manually segmented an anatomical T1 image from the Oxford dataset into six unique tissue types (skin, skull, CSF, GM, WM, Eye) using ITK-SNAP (Yushkevich et al., 2006). Due to the requirement to manually segment each tissue layer, we segmented one complete macaque image. We used this tissue segmentation to create a realistic volumetric head model using SimNIBS 3.2 (Thielscher et al., 2015). The NHP grid was designed and positioned over the lateral prefrontal lobe to mimic the grid used for the human dataset. This region of the brain in NHPs and humans is anatomically comparable (Amiez et al., 2023). The grid consisted of 36 coil locations, organized in a 6 x 6 arrangement with directions spanning left-right and anterior-posterior maintaining a 5 mm spacing illustrated inFigure 1C. We used the Magstim 70 mm Figure-8 coil to simulate 432 electric fields in the NHP model using the same stimulation parameters setup within human studies. We interpolated the simulated fields from the gray matter of the FEM volumetric mesh to the 32k fsLR pial surface of all subjects within the macaque dataset using an ICP algorithm.Figure 1D(left) shows an example of electric field distribution on an NHP 32k fsLR surface.

### 2.7 Subject-level TMS resting-state analysis

We determined an associated seed region for each simulation on the 32k fsLR pial surface for both species. The seed region was determined by thresholding the electric field to greater than 50% of the maximum electric field strength on the pial surface (Opitz et al., 2016). Other electric field threshold values were explored and generated comparable results (Supplementary Fig. 1). In the seed region, we assigned a weight to each node by normalizing the electric field. Example seed regions for a human and a macaque are shown inFigure 1B and D(left). We computed a weighted-average time-series by summing the fMRI time-series at each node and multiplying by the individual node weight from the seed region (Opitz et al., 2016). We correlated the weighted-average time-series with the time-series of each node on the pial surface to calculate a whole-brain functional connectivity map, examples shown inFigure 1C and D(left). To account for the effects of coil location, we calculated correlations using partial correlations, with the mean overall 432 averaged time-series used as a covariate (Opitz et al., 2016).

### 2.8 Parcellation of functional networks

We mapped the Yeo-7 functional networks (Yeo et al., 2011) from FreeSurfer fsaverage surface to the HCP standard fsLR-32k cortical surface using the label-resample command (Glasser et al., 2013). We used this parcellation to identify different functional networks for each participant. The Yeo-7 networks mapped on an example 32k fsLR human surface are shown inFigure 2A. The corresponding Yeo network map on the macaque 32k fsLR surface was generated via a previously established cross-species functional alignment tool (Xu et al., 2020). The NHP Yeo-7 networks mapped on the 32k fsLR NHP surface are shown inFigure 2C.

**Fig. 2.**
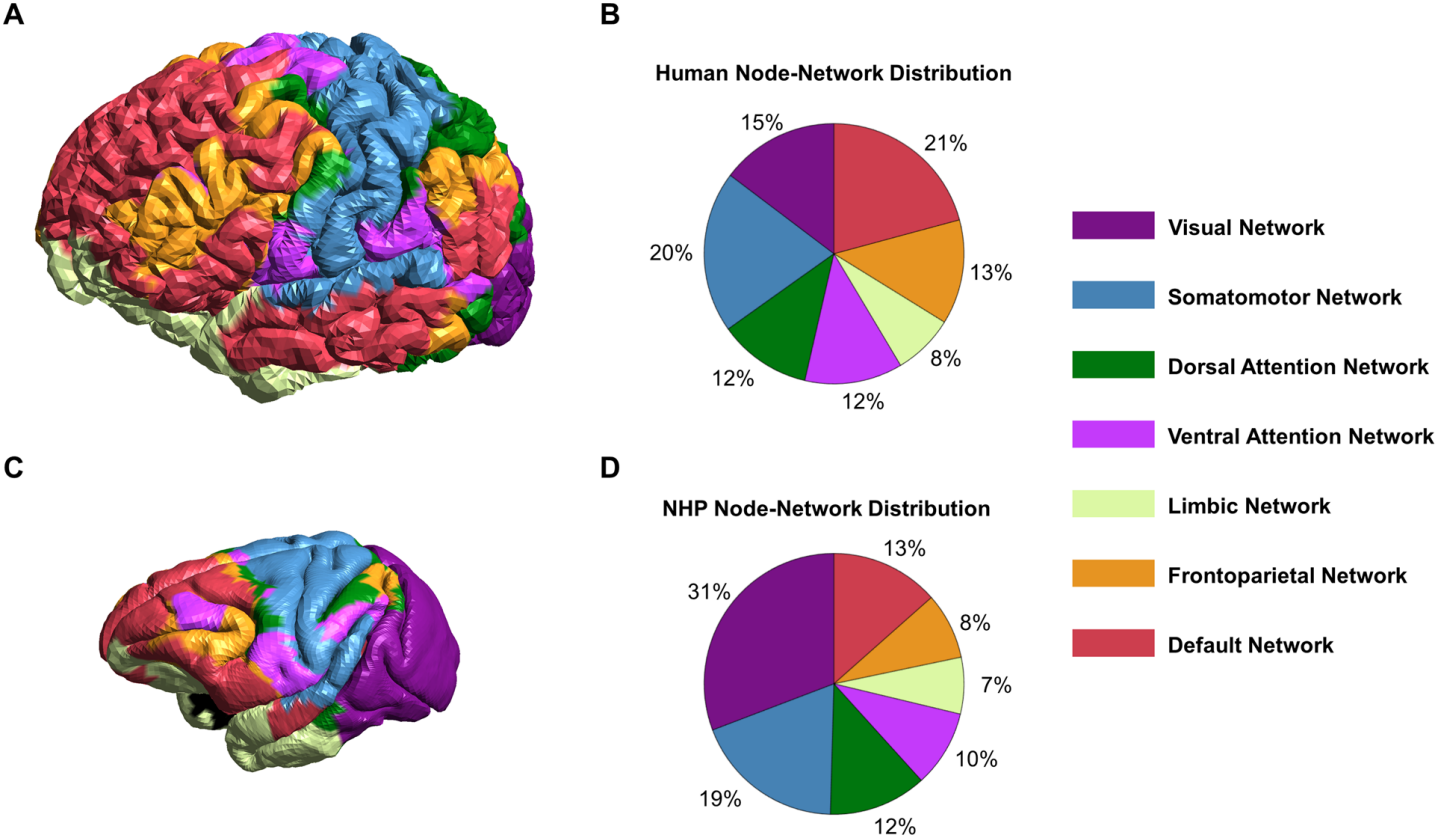
Yeo functional networks across species. The Yeo networks previously established inYeo et al. (2011). (A) Yeo network map on a human 32k fsLR surface and (B) associated functional network node distribution. (C) Yeo network map transferred to the macaque 32k fsLR template surface (Xu et al., 2020) and (D) associated functional network node distribution.

### 2.9 Individual specific TMS functional network analysis

To assign the functional connectivity maps to the Yeo-7 networks, we first applied a sparsity threshold to keep only the top 1% correlation values. Other sparsity thresholds led to similar targeting patterns (Supplementary Figs. 2 and 3). We binarized the functional connectivity maps and calculated the percentage of overlap between the maps for each coil configuration to each Yeo network. Other assignment metrics led to similar network assignments (Supplementary Fig. 4). The targeted functional network evaluations for all participants in the human and macaque datasets were conducted in separate analyses.

### 2.10 Intra-species TMS functional network analysis

**Fig. 3.**
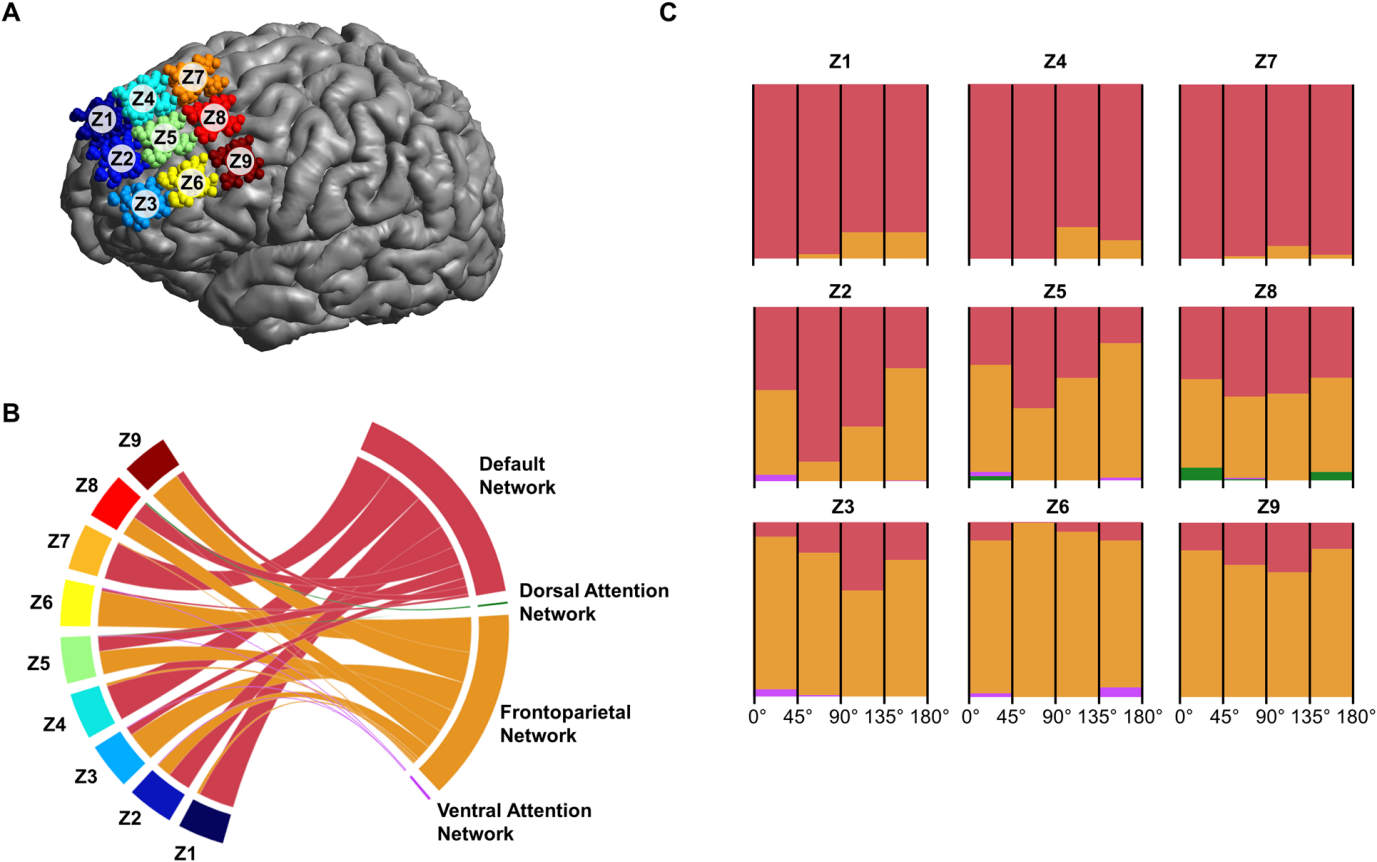
Overlap of TMS networks with Yeo networks in humans. (A) Coil locations grouped into a 3 x 3 zoned grid (Z1-Z9). Each zone represents four stimulation locations per human model. (B) We identified the functional networks overlapping with TMS stimulation in each zone by summarizing all the results of all spatial locations within each zone. In the medial zones (Z1, Z4, Z7), the most targeted network was the Default Network (DN); in the lateral zones (Z3, Z6, Z9), the most targeted network was the Frontoparietal Network (FPN). A transition zone (Z2, Z5, Z8), allowed for stimulating of both FPN and DN. (C) Coil orientations grouped into four 45-degree ranges per grid zone. Within the medial and lateral zones targeting was insensitive to coil orientation while it was sensitive to coil orientation in the transition zone.

To reduce the complexity of the group-level analysis, we assigned the TMS locations across participants to nine zones (Z1-Z9) oriented in a 3 x 3 grid. A single zone area accounted for four coil locations and 12 coil orientations per participant (Fig. 3A). Using these nine zones, we analyzed the overlap of the functional connectivity map with Yeo-7 human and macaque parcellations. We identified each zone’s predicted functional network targeting based on the highest percentage of overlap. In a secondary analysis, we analyzed the effect of TMS coil orientation within each zone. We reduced the simulated coil orientations into groups of 45-degree steps to account for incidental stimulation, resulting in four coil orientations per coil location. The highest overlap coefficients across each zone’s orientation windows were calculated to identify the effect of coil orientation on the predicted TMS-targeted functional networks.

### 2.11 Inter-species TMS functional network analysis

To explain why specific functional networks were more stimulated than others, we identified areas that were preferentially stimulated by TMS across all grid locations in the prefrontal cortex. We calculated an electric field stimulation index (EFSI) that highlights brain regions most often targeted by TMS. This metric quantifies the percentage of nodes within GM that were greater than 50% of the maximum electric field strength across all 432 simulations. To assess the impact of brain gyrification on observed results, we repeated the same analysis for a homogeneous conductivity model (seeSupplementary Fig. 5). The electric field stimulation index was calculated for both species separately.

## 3 Results

### 3.1 Overlap of TMS targeted networks with established functional networks

We first investigated the TMS functional networks for the human participants. We found that the Frontoparietal Network (FPN) and Default Network (DN) dominated the targeted functional networks across stimulation locations (Fig. 3A, B). Specifically, the FPN was most prominent in the lateral zones (Z3, Z6, Z9), while the DN was more stimulated in the medial zones (Z1, Z4, Z7). There was a transition between these two functional networks in the middle zones (Z2, Z5, Z8). We then analyzed the effect of coil orientation on FPN and DN targeting within each zone. Within the lateral and medial zones, network stimulation was predominantly orientation-insensitive. In the transition zone, however, functional network stimulation differed with coil orientation (Fig. 3C). These findings align with previously modeled TMS functional networks in prefrontal brain regions (Opitz et al., 2016).

**Fig. 4.**
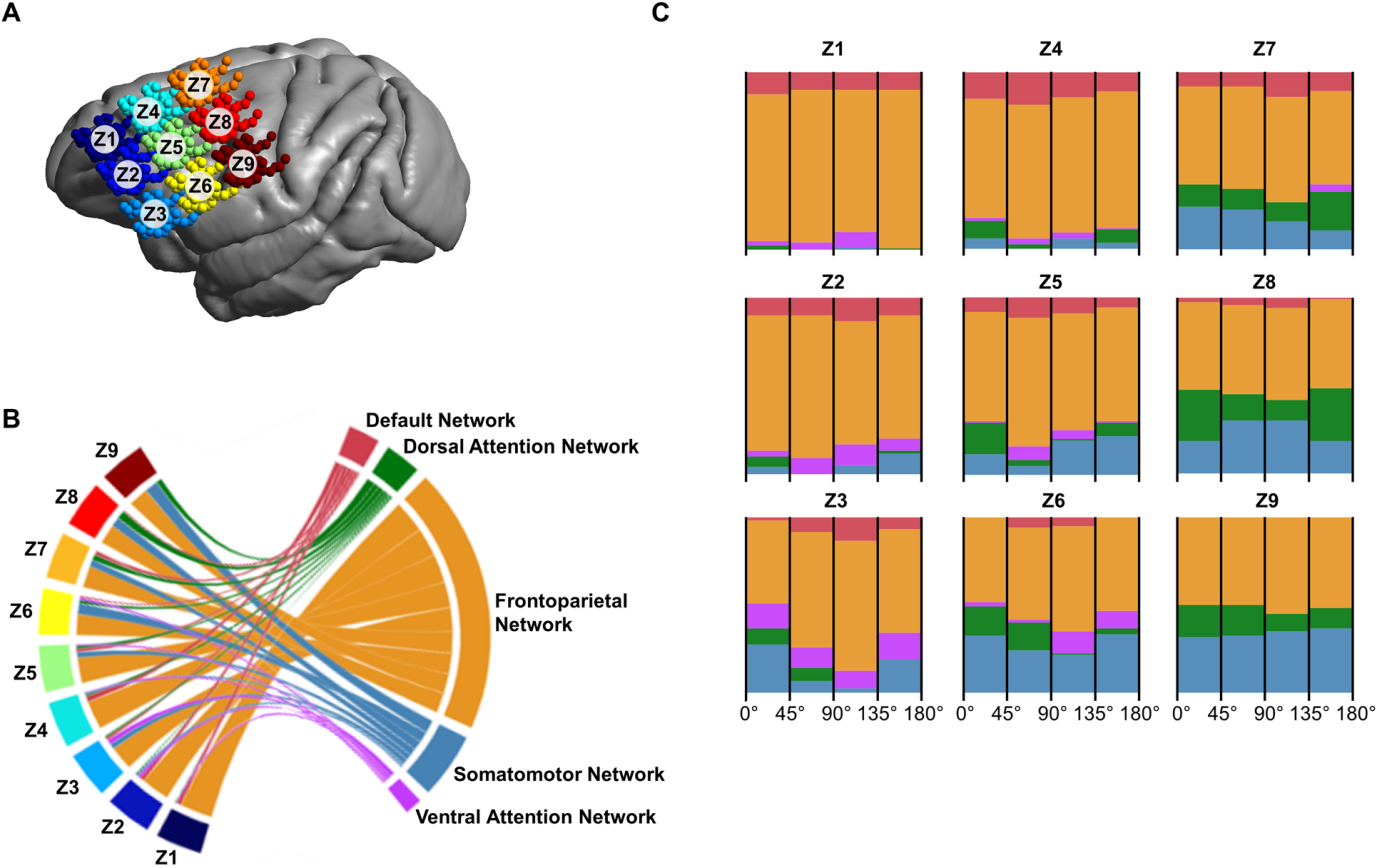
Overlap of TMS networks with Yeo networks in macaques. (A) Coil locations summarized in a 3 x 3 zoned grid, with each section representing four coil locations per NHP model. (B) In the anterior zones (Z1-Z5), the Frontoparietal Network (FPN) was prominently targeted, with instances of Default Network (DN) stimulation in medial locations. The posterior zones (Z6-Z9) are a transitional zone for targeting the FPN or the Somatomotor Network (SN). (C) Coil orientation effects were analyzed by grouping orientations into four 45-degree ranges per zone. In the NHP dataset, TMS network targeting is largely orientation independent.

Next, we invested the functional network targeting with TMS in macaques. When looking at the effect of spatial location, two zones emerged: the anterior zones (Z1-Z5) and the posterior zone (Z6-Z9). Within the anterior zones, there was a preference for the FPN stimulation (Fig. 4A, B). In the posterior zones, we observed a transition from FPN to Somatomotor Network (SN) targeting. Compared to humans, the macaques had increased stimulation of secondary networks, including the DN and Dorsal (DAN) and Ventral Attention Networks (VAN). We then investigated the effect of coil orientation on TMS-network stimulation. In NHPs, targeting was less sensitive to coil orientation and more dependent on spatial location (Fig. 4C).

### 3.2 Cross-species comparison of functional network targeting

**Fig. 5.**
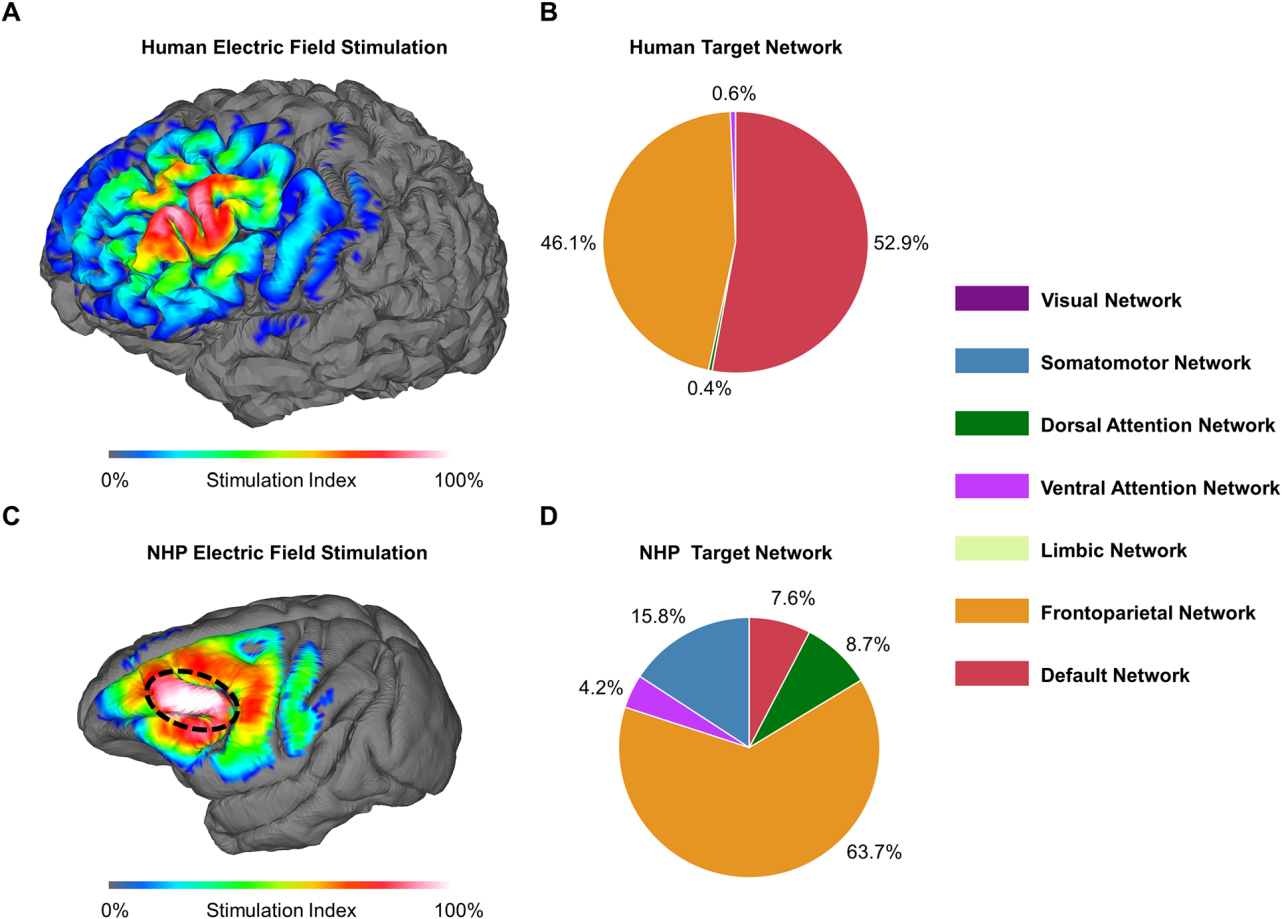
Functional network targets compared across humans and macaques. (A) The electric field activation index is evenly distributed across the stimulation grid in human models, with the highest activation at the grid center. (B) In humans, the Default Network (DN) and Frontoparietal Network (FPN) are targeted roughly equally. (C) In non-human primate (NHP) models, TMS electric fields concentrate along a distinct prefrontal cortex gyral fold. This fold primarily aligns with the FPN. (D) Prefrontal TMS primarily targets the FPN, followed by the Somatomotor Network (SN).

To understand the differences in functional network targeting across species, we investigated which brain regions exhibited the highest electric field stimulation across all 432 simulations by calculating the EFSI. Within the human dataset, the electric field was uniformly spread behind the stimulation grid. The region of highest overlap, 91% of all simulations, coincided with the transitional zone identified in the group-level analysis (Fig. 5A). The two primary functional networks that are stimulated by TMS in the human prefrontal cortex are the DN and the FPN. These are targeted in 53% and 46% of all stimulation conditions, respectively (Fig. 5B). In contrast, other functional networks, including DAN and VAN, exhibit less frequent stimulation.

In all simulation conditions within the NHP models, the electric field strength concentrated along the distinct gyral fold (i.e., area 8a and 46d) in the left prefrontal cortex (Fig. 5C). Stimulation in this brain region occurs in almost all conditions (close to 100%). This behavior is likely due to the surrounding sulci (i.e., the principle sulcus and arcuate sulcus), leading to increased electric field strengths at the CSF-GM interface (Thielscher et al., 2011). In a homogenous conductivity model, the electric field strength is more evenly distributed (seeSupplementary Fig. 5). The prominent prefrontal gyral fold is primarily associated with the FPN within the translated Yeo primate networks (Xu et al., 2020). Thus, the FPN emerged as the dominant target, stimulated in 64% of all TMS configurations (Fig. 5D). Additionally, we found more instances of secondary network targeting in NHP models compared to the human models. The NHP models showed stimulation of the SN (16%), DAN (9%), VAN (4%), and DN (8%).

## 4 Discussion

In this study, we compared TMS functional network targeting in the left prefrontal cortex in humans and macaques. In line with previous work, we found two dominant TMS-network stimulation profiles in the left prefrontal cortex of humans: The frontoparietal network (FPN) and the default network (DN) (Opitz et al., 2016). Specifically, we found targeting of 1) the DN in medial, orientation-insensitive zones, 2) the FPN in lateral orientation-insensitive zones, and 3) DN and FPN in an orientation-sensitive transition zone. In the NHP analysis, we observed that the FPN was predominantly targeted within anterior stimulation zones, which were coil orientation-insensitive. Posterior zones acted as transition zones, stimulating either the FPN or the Somatomotor Network (SN).

Developing protocols to selectively stimulate specific functional networks within the transitional zones is difficult for both species. In a group-level analysis of the human dataset, we observed that the probability of overlapping with the DN or FPN within the transition zone is affected by coil orientation (Fig. 3). At this level, there are no distinctive coil orientations that ensure the targeting of one network over the other. Conducting a group-level analysis on the dataset divided by population demographics (age, sex) did not show significant differences across demographic groups (seeSupplementary Fig. 6). The TMS-network behavior within the transition zones highlights the importance of individualized modeling and analysis. Looking at the transition zone for each individual independently, we can identify individual stimulation schemes to target the DN or FPN (seeSupplementary Figs. 9,10,11). The variations observed across the human dataset can be attributed to the compounding effect of individualities such as neuroanatomy and intrinsic network behavior.

The need for individualized targeting protocols also extends to NHP models. At the group level, TMS-network patterns in the posterior transition zone are insensitive to coil orientation (Fig. 4). Despite this, there were no distinct coil configurations that could guarantee the stimulation of a specific network. NHPs have individualities beyond neuroanatomy and intrinsic brain behavior that can impact TMS-network targeting. These factors can include sex, age, weight, and tissue composition. The NHPs included in the study dataset were all male. While this does eliminate the individualities associated with sex on our observations, this could be an interesting point for future studies. Subdividing the dataset to analyze the effect of age and weight on TMS-network stimulation did not eliminate the observed complexity (seeSupplementary Fig. 7). In NHPs, the tissue composition of the head (i.e., the skin, muscle, and skull thickness) can vary greatly. These tissues impact the distance between the coil and the GM surface of the brain. The strength of the electric field induced on the GM surface is inversely related to the distance between the coil and the GM surface. However, the center of stimulation underneath the TMS coil is largely stable for differences in non-brain tissues (Alekseichuk et al., 2019;Mantell et al., 2023). For practical applications, the TMS intensity can be adapted to reach an effective electric field strength in the brain. The TMS-network targeting behavior of the NHPs emphasizes the importance of creating individualized models and stimulation protocols. While some individualities can be generalized, the unique interplay between individual neuroanatomy and intrinsic brain behavior cannot.

An important distinction between humans and NHPs is the ability to target the DN with TMS. We found that in macaques, targeting the corresponding DN in the left prefrontal lobe is notably more challenging than in humans. One reason for this observation is the difference in brain gyrification across species. Compared to humans, the macaque NHP cortical surface is less gyrated (Hofman, 2014;Van Essen & Dierker, 2007). Specifically, within the prefrontal cortex, the human brain is highly gyrated while the macaque brain has one prominent gyrus.

Gyrification patterns play an influential role in the properties of the TMS-induced electric field. TMS electric fields are enhanced in GM when currents are crossing the CSF-GM interface (Miranda et al., 2007;Thielscher et al., 2011). The anatomical features, including gyri, affect which regions get preferentially stimulated across the investigated TMS coil locations (Fig. 5). In humans, we found preferential targeting of the DN-FPN in the transition zone and evenly distributed electric field stimulation overlaying each specific network. In comparison, we found preferential stimulation of the dominant cortical fold primarily corresponding to the FPN in macaques. Thus, as observed in the TMS-functional network, targeting across species is affected by differences in brain gyrification.

The NHPs and humans exhibit differences in secondary functional network stimulation. The investigated TMS coil locations equally target two distinct networks (DN and FPN) across the human models. In NHP models, however, equivalent coil locations preferentially stimulated the FPN network. Moreover, NHPs exhibited TMS electric field overlap with a higher number of secondary networks than humans. These secondary networks include the dorsal attention network, somatomotor network, ventral attention network, and DN. The increased overlap of secondary networks could be the effect of widespread electric field distribution on the NHP cortical surface. The NHP brain models are comparatively smaller than human brain models. Notably, the cortical gray matter of the human prefrontal cortex exceeds that of the macaques by up to 1.9-fold (Donahue et al., 2018). Smaller brain size results in more electric field distribution relative to the surface area (Alekseichuk et al., 2019). In addition to being smaller, the networks are closer together on the NHP cortical surface (Fig. 2). These factors could lead to the incidental stimulation of other networks outside the targeted ones. One method to reduce incidental stimulation is using a smaller TMS coil. Smaller coils result in increased focality but reduced electric field strength. In our simulations, using a 25 mm Figure-8 Magstim coil did not result in significant targeting differences when compared to the 70 mm coil (seeSupplementary Fig. 8) for either species.

The prefrontal cortex in humans is remarkably expanded, resulting in pronounced connections to multimodal areas and greater network modularity (Garin et al., 2022;Liu et al., 2019;Mantini et al., 2011). DN stands out as the network exhibiting the most significant differences in both structure and function between humans and NHPs (Xu et al., 2020). Although DN appears to be present in NHPs in the medial frontal and the posterior cingulate cortex, the lateral prefrontal cortex in macaque likely plays a role in attention and executive functions (Bahmani et al., 2019;Bullock et al., 2017;Petrides et al., 2012). Of note, the fMRI data for NHPs were acquired during anesthesia, which has been demonstrated to have a significant impact on brain activity and network characterizations (Aksenov et al., 2015;Hudetz, 2012;Hutchison et al., 2013;Jovellar & Doudet, 2019;Xu et al., 2018;Zhang, 2022). Generally, anesthesia reduces overall functional activation and connectivity strength, particularly in long-distance connectivity (Leung et al., 2014;Xu et al., 2019). Thalamocortical connectivity is commonly suppressed and high-order networks, such as the default model and frontoparietal networks, tend to be disrupted. These specific effects on functional connectivity also depend on the type and dosage of the anesthesia agent used (Hutchison et al., 2014;Paasonen et al., 2018). Therefore, our study may not fully capture the nuanced effects of anesthesia on the networks in macaques, suggesting the need for direct comparisons with awake data in the future.

The comparison of TMS functional networks between humans and NHPs did not reveal a direct one-to-one mapping across species in the prefrontal cortex. Variability within and across species led to different functional networks targeted by TMS and highlights the challenges of cross-species translation. Our comprehensive study of TMS functional network stimulation can, thus, inform future translational efforts. Prefrontal TMS is relevant in the treatment of various neuropsychiatric disorders such as depression. Brain stimulation therapies targeting functional networks within this region are a promising tool to modulate neural circuits affected by specific disorders (Siddiqi et al., 2020). NHPs offer a unique opportunity to validate and refine TMS-network strategies. Our cross-species network comparison can guide these efforts to help improve translational efforts in NHPs. Future experimental work is needed to validate the predictions made by our network targeting approach in both humans and NHPs.

## Supplementary Material

Supplementary Material

## Data Availability

The human data used in this study are publicly available via the Human Connectome Project:http://www.humanconnectomeproject.org/ The non-human primate data used in this study are publicly available on the PRIMatE Data Exchange:https://fcon_1000.projects.nitrc.org/indi/indiPRIME.html

## References

[b1] Aksenov , D. P. , Li , L. , Miller , M. J. , Iordanescu , G. , & Wyrwicz , A. M. ( 2015 ). Effects of anesthesia on BOLD signal and neuronal activity in the somatosensory cortex . Journal of Cerebral Blood Flow & Metabolism , 35 ( 11 ), 1819 – 1826 . 10.1038/jcbfm.2015.130 26104288 PMC4635237

[b2] Alekseichuk , I. , Mantell , K. , Shirinpour , S. , & Opitz , A. ( 2019 ). Comparative modeling of transcranial magnetic and electric stimulation in mouse, monkey, and human . NeuroImage , 194 , 136 – 148 . 10.1016/j.neuroimage.2019.03.044 30910725 PMC6536349

[b3] Amiez , C. , Kostopoulos , P. , Champod , A.-S. , & Petrides , M. ( 2006 ). Local morphology predicts functional organization of the dorsal premotor region in the human brain . The Journal of Neuroscience , 26 ( 10 ), 2724 – 2731 . 10.1523/JNEUROSCI.4739-05.2006 16525051 PMC6675158

[b4] Amiez , C. , Sallet , J. , Giacometti , C. , Verstraete , C. , Gandaux , C. , Morel-Latour , V. , Meguerditchian , A. , Hadj-Bouziane , F. , Ben Hamed , S. , Hopkins , W. D. , Procyk , E. , Wilson , C. R. E. , & Petrides , M. ( 2023 ). A revised perspective on the evolution of the lateral frontal cortex in primates . Science Advances , 9 ( 20 ), eadf9445 . 10.1126/sciadv.adf9445 37205762 PMC10198639

[b5] Ardesch , D. J. , Scholtens , L. H. , Li , L. , Preuss , T. M. , Rilling , J. K. , & van den Heuvel , M. P. ( 2019 ). Evolutionary expansion of connectivity between multimodal association areas in the human brain compared with chimpanzees . Proceedings of the National Academy of Sciences of the United States of America , 116 ( 14 ), 7101 – 7106 . 10.1073/pnas.1818512116 30886094 PMC6452697

[b6] Avery , D. H. , Holtzheimer , P. E. , Fawaz , W. , Russo , J. , Neumaier , J. , Dunner , D. L. , Haynor , D. R. , Claypoole , K. H. , Wajdik , C. , & Roy-Byrne , P. ( 2006 ). A controlled study of repetitive transcranial magnetic stimulation in medication-resistant major depression . Biological Psychiatry , 59 ( 2 ), 187 – 194 . 10.1016/j.biopsych.2005.07.003 16139808

[b7] Bahmani , Z. , Clark , K. , Merrikhi , Y. , Mueller , A. , Pettine , W. , Isabel Vanegas , M. , Moore , T. , & Noudoost , B. ( 2019 ). Prefrontal contributions to attention and working memory . In T. Hodgson (Ed.), Processes of visuospatial attention and working memory (pp. 129 – 153 ). Springer International Publishing . 10.1007/7854_2018_74 PMC6689265 30739308

[b8] Bullock , K. R. , Pieper , F. , Sachs , A. J. , & Martinez-Trujillo , J. C. ( 2017 ). Visual and presaccadic activity in area 8Ar of the macaque monkey lateral prefrontal cortex . Journal of Neurophysiology , 118 ( 1 ), 15 – 28 . 10.1152/jn.00278.2016 28298302 PMC5494359

[b9] de Lima-Pardini , A. C. , Mikhail , Y. , Dominguez-Vargas , A.-U. , Dancause , N. , & Scott , S. H. ( 2023 ). Transcranial magnetic stimulation in non-human primates: A systematic review . Neuroscience & Biobehavioral Reviews , 152 , 105273 . 10.1016/j.neubiorev.2023.105273 37315659

[b10] Deng , Z.-D. , Lisanby , S. H. , & Peterchev , A. V. ( 2013 ). Electric field depth–focality tradeoff in transcranial magnetic stimulation: Simulation comparison of 50 coil designs . Brain Stimulation , 6 ( 1 ), 1 – 13 . 10.1016/j.brs.2012.02.005 22483681 PMC3568257

[b11] Donahue , C. J. , Glasser , M. F. , Preuss , T. M. , Rilling , J. K. , & Van Essen , D. C. ( 2018 ). Quantitative assessment of prefrontal cortex in humans relative to nonhuman primates . Proceedings of the National Academy of Sciences of the United States of America , 115 ( 22 ), E5183 – E5192 . 10.1073/pnas.1721653115 29739891 PMC5984508

[b12] Fox , M. D. , Halko , M. A. , Eldaief , M. C. , & Pascual-Leone , A. ( 2012 ). Measuring and manipulating brain connectivity with resting state functional connectivity magnetic resonance imaging (fcMRI) and transcranial magnetic stimulation (TMS) . Neuroimage , 62 ( 4 ), 2232 – 2243 . 10.1016/j.neuroimage.2012.03.035 22465297 PMC3518426

[b13] Friedman , N. P. , & Robbins , T. W. ( 2022 ). The role of prefrontal cortex in cognitive control and executive function . Neuropsychopharmacology , 47 ( 1 ), 72 – 89 . 10.1038/s41386-021-01132-0 34408280 PMC8617292

[b14] Garin , C. M. , Hori , Y. , Everling , S. , Whitlow , C. T. , Calabro , F. J. , Luna , B. , Froesel , M. , Gacoin , M. , Ben Hamed , S. , Dhenain , M. , & Constantinidis , C. ( 2022 ). An evolutionary gap in primate default mode network organization . Cell Reports , 39 ( 2 ), 110669 . 10.1016/j.celrep.2022.110669 35417698 PMC9088817

[b15] George , M. S. , Lisanby , S. H. , Avery , D. , McDonald , W. M. , Durkalski , V. , Pavlicova , M. , Anderson , B. , Nahas , Z. , Bulow , P. , Zarkowski , P. , Holtzheimer , P. E. , III , Schwartz, T. , & Sackeim , H. A. ( 2010 ). Daily left prefrontal transcranial magnetic stimulation therapy for major depressive disorder: A sham-controlled randomized trial . Archives of General Psychiatry , 67 ( 5 ), 507 – 516 . 10.1001/archgenpsychiatry.2010.46 20439832

[b16] George , M. S. , Wassermann , E. M. , Williams , W. A. , Callahan , A. , Ketter , T. A. , Basser , P. , Hallett , M. , & Post , R. M. ( 1995 ). Daily repetitive transcranial magnetic stimulation (rTMS) improves mood in depression . Neuroreport , 6 ( 14 ), 1853 – 1856 . 10.1097/00001756-199510020-00008 8547583

[b17] Glasser , M. F. , Sotiropoulos , S. N. , Wilson , J. A. , Coalson , T. S. , Fischl , B. , Andersson , J. L. , Xu , J. , Jbabdi , S. , Webster , M. , Polimeni , J. R. , Van Essen , D. C. , Jenkinson , M. , & WU-Minn HCP Consortium . ( 2013 ). The minimal preprocessing pipelines for the Human Connectome Project . NeuroImage , 80 , 105 – 124 . 10.1016/j.neuroimage.2013.04.127 23668970 PMC3720813

[b18] Goulas , A. , Uylings , H. B. M. , & Stiers , P. ( 2012 ). Unravelling the intrinsic functional organization of the human lateral frontal cortex: A parcellation scheme based on resting state fMRI . Journal of Neuroscience , 32 ( 30 ), 10238 – 10252 . 10.1523/JNEUROSCI.5852-11.2012 22836258 PMC6703746

[b19] Gratton , C. , Kraus , B. T. , Greene , D. J. , Gordon , E. M. , Laumann , T. O. , Nelson , S. M. , Dosenbach , N. U. F. , & Petersen , S. E. ( 2020 ). Defining individual-specific functional neuroanatomy for precision psychiatry . Biological Psychiatry , 88 ( 1 ), 28 – 39 . 10.1016/j.biopsych.2019.10.026 31916942 PMC7203002

[b20] Hamada , M. , Murase , N. , Hasan , A. , Balaratnam , M. , & Rothwell , J. C. ( 2013 ). The role of interneuron networks in driving human motor cortical plasticity . Cerebral Cortex , 23 ( 7 ), 1593 – 1605 . 10.1093/cercor/bhs147 22661405

[b21] Hanlon , C. A. , Czoty , P. W. , Smith , H. R. , Epperly , P. M. , & Galbo , L. K. ( 2021 ). Cortical excitability in a nonhuman primate model of TMS . Brain Stimulation , 14 ( 1 ), 19 – 21 . 10.1016/j.brs.2020.10.008 33099045 PMC7934343

[b22] Herbsman , T. , Avery , D. , Ramsey , D. , Holtzheimer , P. , Wadjik , C. , Hardaway , F. , Haynor , D. , George , M. S. , & Nahas , Z. ( 2009 ). More lateral and anterior prefrontal coil location is associated with better repetitive transcranial magnetic stimulation antidepressant response . Biological Psychiatry , 66 ( 5 ), 509 – 515 . 10.1016/j.biopsych.2009.04.034 19545855

[b23] Herwig , U. , Padberg , F. , Unger , J. , Spitzer , M. , & Schönfeldt-Lecuona , C. ( 2001 ). Transcranial magnetic stimulation in therapy studies: Examination of the reliability of “standard” coil positioning by neuronavigation . Biological Psychiatry , 50 ( 1 ), 58 – 61 . 10.1016/S0006-3223(01)01153-2 11457424

[b24] Hofman , M. ( 2014 ). Evolution of the human brain: When bigger is better . Frontiers in Neuroanatomy , 8 , 15 . 10.3389/fnana.2014.00015 24723857 PMC3973910

[b25] Hudetz , A. G. ( 2012 ). General anesthesia and human brain connectivity . Brain Connectivity , 2 ( 6 ), 291 – 302 . 10.1089/brain.2012.0107 23153273 PMC3621592

[b26] Hutchison , R. M. , Hutchison , M. , Manning , K. Y. , Menon , R. S. , & Everling , S. ( 2014 ). Isoflurane induces dose-dependent alterations in the cortical connectivity profiles and dynamic properties of the brain’s functional architecture . Human Brain Mapping , 35 ( 12 ), 5754 – 5775 . 10.1002/hbm.22583 25044934 PMC6869297

[b27] Hutchison , R. M. , Womelsdorf , T. , Gati , J. S. , Everling , S. , & Menon , R. S. ( 2013 ). Resting-state networks show dynamic functional connectivity in awake humans and anesthetized macaques . Human Brain Mapping , 34 ( 9 ), 2154 – 2177 . 10.1002/hbm.22058 22438275 PMC6870538

[b28] Jovellar , D. B. , & Doudet , D. J. ( 2019 ). fMRI in non-human primate: A review on factors that can affect interpretation and dynamic causal modeling application . Frontiers in Neuroscience , 13 , 973 . 10.3389/fnins.2019.00973 31619951 PMC6759819

[b29] Laumann , T. O. , Gordon , E. M. , Adeyemo , B. , Snyder , A. Z. , Joo , S. J. , Chen , M.-Y. , Gilmore , A. W. , McDermott , K. B. , Nelson , S. M. , Dosenbach , N. U. F. , Schlaggar , B. L. , Mumford , J. A. , Poldrack , R. A. , & Petersen , S. E. ( 2015 ). Functional system and areal organization of a highly sampled individual human brain . Neuron , 87 ( 3 ), 657 – 670 . 10.1016/j.neuron.2015.06.037 26212711 PMC4642864

[b30] Lear , A. , Baker , S. N. , Clarke , H. F. , Roberts , A. C. , Schmid , M. C. , & Jarrett , W. ( 2022 ). Understanding them to understand ourselves: The importance of NHP research for translational neuroscience . Current Research in Neurobiology , 3 , 100049 . 10.1016/j.crneur.2022.100049 36518342 PMC9743051

[b31] Lefaucheur , J.-P. , André-Obadia , N. , Antal , A. , Ayache , S. S. , Baeken , C. , Benninger , D. H. , Cantello , R. M. , Cincotta , M. , de Carvalho , M. , De Ridder , D. , Devanne , H. , Di Lazzaro , V. , Filipović , S. R. , Hummel , F. C. , Jääskeläinen , S. K. , Kimiskidis , V. K. , Koch , G. , Langguth , B. , Nyffeler , T. , … Garcia-Larrea , L. ( 2014 ). Evidence-based guidelines on the therapeutic use of repetitive transcranial magnetic stimulation (rTMS) . Clinical Neurophysiology , 125 ( 11 ), 2150 – 2206 . 10.1016/j.clinph.2014.05.021 25034472

[b32] Leung , L. S. , Luo , T. , Ma , J. , & Herrick , I. ( 2014 ). Brain areas that influence general anesthesia . Progress in Neurobiology , 122 , 24 – 44 . 10.1016/j.pneurobio.2014.08.001 25172271

[b33] Liu , C. , Yen , C. C.-C. , Szczupak , D. , Ye , F. Q. , Leopold , D. A. , & Silva , A. C. ( 2019 ). Anatomical and functional investigation of the marmoset default mode network . Nature Communications , 10 , 1975 . 10.1038/s41467-019-09813-7 PMC6488610 31036814

[b34] López-Alonso , V. , Cheeran , B. , Río-Rodríguez , D. , & Fernández-del-Olmo , M. ( 2014 ). Inter-individual variability in response to non-invasive brain stimulation paradigms . Brain Stimulation , 7 ( 3 ), 372 – 380 . 10.1016/j.brs.2014.02.004 24630849

[b35] Mantell , K. E. , Perera , N. D. , Shirinpour , S. , Puonti , O. , Xu , T. , Zimmermann , J. , Falchier , A. , Heilbronner , S. R. , Thielscher , A. , & Opitz , A. ( 2023 ). Anatomical details affect electric field predictions for non-invasive brain stimulation in non-human primates . NeuroImage , 279 , 120343 . 10.1016/j.neuroimage.2023.120343 37619797 PMC10961993

[b36] Mantini , D. , Gerits , A. , Nelissen , K. , Durand , J.-B. , Joly , O. , Simone , L. , Sawamura , H. , Wardak , C. , Orban , G. A. , Buckner , R. L. , & Vanduffel , W. ( 2011 ). Default mode of brain function in monkeys . Journal of Neuroscience , 31 ( 36 ), 12954 – 12962 . 10.1523/JNEUROSCI.2318-11.2011 21900574 PMC3686636

[b37] Margulies , D. S. , & Petrides , M. ( 2013 ). Distinct parietal and temporal connectivity profiles of ventrolateral frontal areas involved in language production . Journal of Neuroscience , 33 ( 42 ), 16846 – 16852 . 10.1523/JNEUROSCI.2259-13.2013 24133284 PMC6618526

[b38] Mars , R. B. , Sotiropoulos , S. N. , Passingham , R. E. , Sallet , J. , Verhagen , L. , Khrapitchev , A. A. , Sibson , N. , & Jbabdi , S. ( 2018 ). Whole brain comparative anatomy using connectivity blueprints . eLife , 7 , e35237 . 10.7554/eLife.35237 29749930 PMC5984034

[b39] Milham , M. P. , Ai , L. , Koo , B. , Xu , T. , Amiez , C. , Balezeau , F. , Baxter , M. G. , Blezer , E. L. A. , Brochier , T. , Chen , A. , Croxson , P. L. , Damatac , C. G. , Dehaene , S. , Everling , S. , Fair , D. A. , Fleysher , L. , Freiwald , W. , Froudist-Walsh , S. , Griffiths , T. D. , … Schroeder , C. E. ( 2018 ). An open resource for non-human primate imaging . Neuron , 100 ( 1 ), 61.e2 – 74.e2 . 10.1016/j.neuron.2018.08.039 30269990 PMC6231397

[b40] Miranda , P. C. , Correia , L. , Salvador , R. , & Basser , P. J. ( 2007 ). Tissue heterogeneity as a mechanism for localized neural stimulation by applied electric fields . Physics in Medicine and Biology , 52 ( 18 ), 5603 – 5617 . 10.1088/0031-9155/52/18/009 17804884

[b41] Mueller , J. K. , Grigsby , E. M. , Prevosto , V. , Petraglia , F. W. , Rao , H. , Deng , Z.-D. , Peterchev , A. V. , Sommer , M. A. , Egner , T. , Platt , M. L. , & Grill , W. M. ( 2014 ). Simultaneous transcranial magnetic stimulation and single-neuron recording in alert non-human primates . Nature Neuroscience , 17 ( 8 ), 1130 – 1136 . 10.1038/nn.3751 24974797 PMC4115015

[b42] Noonan , M. P. , Sallet , J. , Mars , R. B. , Neubert , F. X. , O’Reilly , J. X. , Andersson , J. L. , Mitchell , A. S. , Bell , A. H. , Miller , K. L. , & Rushworth , M. F. S. ( 2014 ). A neural circuit covarying with social hierarchy in macaques . PLoS Biology , 12 ( 9 ), e1001940 . 10.1371/journal.pbio.1001940 25180883 PMC4151964

[b43] Oathes , D. J. , Zimmerman , J. P. , Duprat , R. , Japp , S. S. , Scully , M. , Rosenberg , B. M. , Flounders , M. W. , Long , H. , Deluisi , J. A. , Elliott , M. , Shandler , G. , Shinohara , R. T. , & Linn , K. A. ( 2021 ). Resting fMRI guided TMS results in subcortical and brain network modulation indexed by interleaved TMS/fMRI . Experimental Brain Research , 239 ( 4 ), 1165 – 1178 . 10.1007/s00221-021-06036-5 33560448 PMC8521442

[b44] Opitz , A. , Fox , M. D. , Craddock , R. C. , Colcombe , S. , & Milham , M. P. ( 2016 ). An integrated framework for targeting functional networks via transcranial magnetic stimulation . NeuroImage , 127 , 86 – 96 . 10.1016/j.neuroimage.2015.11.040 26608241 PMC4836057

[b45] Opitz , A. , Windhoff , M. , Heidemann , R. M. , Turner , R. , & Thielscher , A. ( 2011 ). How the brain tissue shapes the electric field induced by transcranial magnetic stimulation . NeuroImage , 58 ( 3 ), 849 – 859 . 10.1016/j.neuroimage.2011.06.069 21749927

[b46] Paasonen , J. , Stenroos , P. , Salo , R. A. , Kiviniemi , V. , & Gröhn , O. ( 2018 ). Functional connectivity under six anesthesia protocols and the awake condition in rat brain . NeuroImage , 172 , 9 – 20 . 10.1016/j.neuroimage.2018.01.014 29414498

[b47] Pascual-Leone , A. , Rubio , B. , Pallardó , F. , & Catalá , M. D. ( 1996 ). Rapid-rate transcranial magnetic stimulation of left dorsolateral prefrontal cortex in drug-resistant depression . The Lancet , 348 ( 9022 ), 233 – 237 . 10.1016/S0140-6736(96)01219-6 8684201

[b48] Perera , N. D. , Alekseichuk , I. , Shirinpour , S. , Wischnewski , M. , Linn , G. , Masiello , K. , Butler , B. , Russ , B. E. , Schroeder , C. E. , Falchier , A. , & Opitz , A. ( 2023 ). Dissociation of centrally and peripherally induced transcranial magnetic stimulation effects in nonhuman primates . Journal of Neuroscience , 43 ( 50 ), 8649 – 8662 . 10.1523/JNEUROSCI.1016-23.2023 37852789 PMC10727178

[b49] Petrides , M. , Tomaiuolo , F. , Yeterian , E. H. , & Pandya , D. N. ( 2012 ). The prefrontal cortex: Comparative architectonic organization in the human and the macaque monkey brains . Cortex , 48 ( 1 ), 46 – 57 . 10.1016/j.cortex.2011.07.002 21872854

[b50] Power , J. D. , Cohen , A. L. , Nelson , S. M. , Wig , G. S. , Barnes , K. A. , Church , J. A. , Vogel , A. C. , Laumann , T. O. , Miezin , F. M. , Schlaggar , B. L. , & Petersen , S. E. ( 2011 ). Functional network organization of the human brain . Neuron , 72 ( 4 ), 665 – 678 . 10.1016/j.neuron.2011.09.006 22099467 PMC3222858

[b51] Rizvi , S. , & Khan , A. M. ( 2019 ). Use of transcranial magnetic stimulation for depression . Cureus , 11 ( 5 ), e4736 . 10.7759/cureus.4736 31355095 PMC6649915

[b52] Romero , M. C. , Davare , M. , Armendariz , M. , & Janssen , P. ( 2019 ). Neural basis of transcranial magnetic stimulation at the single-cell level . Nature Communication , 2019 , 405753 – 405753 . 10.1101/405753 PMC6572776 31201331

[b53] Rossi , S. , Hallett , M. , Rossini , P. M. , Pascual-Leone , A. , & Safety of TMS Consensus Group . ( 2009 ). Safety, ethical considerations, and application guidelines for the use of transcranial magnetic stimulation in clinical practice and research . Clinical Neurophysiology: Official Journal of the International Federation of Clinical Neurophysiology , 120 ( 12 ), 2008 – 2039 . 10.1016/j.clinph.2009.08.016 19833552 PMC3260536

[b54] Rusinkiewicz , S. , & Levoy , M. ( 2001 ). Efficient variants of the ICP algorithm . In Proceedings Third International Conference on 3-D Digital Imaging and Modeling (pp. 145 – 152 ). IEEE . 10.1109/IM.2001.924423

[b55] Schutter , D. J. L. G. ( 2009 ). Antidepressant efficacy of high-frequency transcranial magnetic stimulation over the left dorsolateral prefrontal cortex in double-blind sham-controlled designs: A meta-analysis . Psychological Medicine , 39 ( 1 ), 65 – 75 . 10.1017/S0033291708003462 18447962

[b56] Siddiqi , S. H. , Taylor , S. F. , Cooke , D. , Pascual-Leone , A. , George , M. S. , & Fox , M. D. ( 2020 ). Distinct symptom-specific treatment targets for circuit-based neuromodulation . American Journal of Psychiatry , 177 ( 5 ), 435 – 446 . 10.1176/appi.ajp.2019.19090915 32160765 PMC8396109

[b57] Smith , S. M. , Andersson , J. , Auerbach , E. J. , Beckmann , C. F. , Bijsterbosch , J. , Douaud , G. , Duff , E. , Feinberg , D. A. , Griffanti , L. , Harms , M. P. , Kelly , M. , Laumann , T. , Miller , K. L. , Moeller , S. , Petersen , S. , Power , J. , Salimi-Khorshidi , G. , Snyder , A. Z. , Vu , A. , … Glasser , M. F. ( 2013 ). Resting-state fMRI in the Human Connectome Project . NeuroImage , 80 , 144 – 168 . 10.1016/j.neuroimage.2013.05.039 23702415 PMC3720828

[b58] Thielscher , A. , Antunes , A. , & Saturnino , G. B. ( 2015 ). Field modeling for transcranial magnetic stimulation: A useful tool to understand the physiological effects of TMS? 2015 37th Annual International Conference of the IEEE Engineering in Medicine and Biology Society , 2015 , 222 – 225 . 10.1109/EMBC.2015.7318340 26736240

[b59] Thielscher , A. , Opitz , A. , & Windhoff , M. ( 2011 ). Impact of the gyral geometry on the electric field induced by transcranial magnetic stimulation . NeuroImage , 54 ( 1 ), 234 – 243 . 10.1016/j.neuroimage.2010.07.061 20682353

[b60] van den Heuvel , M. P. , Ardesch , D. J. , Scholtens , L. H. , de Lange , S. C. , van Haren , N. E. M. , Sommer , I. E. C. , Dannlowski , U. , Repple , J. , Preuss , T. M. , Hopkins , W. D. , & Rilling , J. K. ( 2023 ). Human and chimpanzee shared and divergent neurobiological systems for general and specific cognitive brain functions . Proceedings of the National Academy of Sciences of the United States of America , 120 ( 22 ), e2218565120 . 10.1073/pnas.2218565120 37216540 PMC10235977

[b61] Van Essen , D. C. ( 2004 ). Surface-based approaches to spatial localization and registration in primate cerebral cortex . NeuroImage , 23 ( Suppl. 1 ), S97 – S107 . 10.1016/j.neuroimage.2004.07.024 15501104

[b62] Van Essen , D. C. , & Dierker , D. L. ( 2007 ). Surface-based and probabilistic atlases of primate cerebral cortex . Neuron , 56 ( 2 ), 209 – 225 . 10.1016/j.neuron.2007.10.015 17964241

[b63] Van Essen , D. C. , Ugurbil , K. , Auerbach , E. , Barch , D. , Behrens , T. E. J. , Bucholz , R. , Chang , A. , Chen , L. , Corbetta , M. , Curtiss , S. W. , Della Penna , S. , Feinberg , D. , Glasser , M. F. , Harel , N. , Heath , A. C. , Larson-Prior , L. , Marcus , D. , Michalareas , G. , Moeller , S. , … WU-Minn HCP Consortium . ( 2012 ). The Human Connectome Project: A data acquisition perspective . NeuroImage , 62 ( 4 ), 2222 – 2231 . 10.1016/j.neuroimage.2012.02.018 22366334 PMC3606888

[b64] Wassermann , E. M. , & Lisanby , S. H. ( 2001 ). Therapeutic application of repetitive transcranial magnetic stimulation: A review . Clinical Neurophysiology , 112 ( 8 ), 1367 – 1377 . 10.1016/S1388-2457(01)00585-5 11459676

[b65] Windhoff , M. , Opitz , A. , & Thielscher , A. ( 2013 ). Electric field calculations in brain stimulation based on finite elements: An optimized processing pipeline for the generation and usage of accurate individual head models . Human Brain Mapping , 34 ( 4 ), 923 – 935 . 10.1002/hbm.21479 22109746 PMC6870291

[b66] WU_Minn , H. C. P. ( 2017 ). 1200 Subject data release reference manual . https://www.humanconnectome.org/storage/app/media/documentation/s1200/HCP_S1200_Release_Reference_Manual.pdf

[b67] Xu , T. , Falchier , A. , Sullivan , E. L. , Linn , G. , Ramirez , J. S. B. , Ross , D. , Feczko , E. , Opitz , A. , Bagley , J. , Sturgeon , D. , Earl , E. , Miranda-Domínguez , O. , Perrone , A. , Craddock , R. C. , Schroeder , C. E. , Colcombe , S. , Fair , D. A. , & Milham , M. P. ( 2018 ). Delineating the macroscale areal organization of the macaque cortex in vivo . Cell Reports , 23 ( 2 ), 429 – 441 . 10.1016/j.celrep.2018.03.049 29642002 PMC6157013

[b68] Xu , T. , Nenning , K.-H. , Schwartz , E. , Hong , S.-J. , Vogelstein , J. T. , Goulas , A. , Fair , D. A. , Schroeder , C. E. , Margulies , D. S. , Smallwood , J. , Milham , M. P. , & Langs , G. ( 2020 ). Cross-species functional alignment reveals evolutionary hierarchy within the connectome . NeuroImage , 223 , 117346 . 10.1016/j.neuroimage.2020.117346 32916286 PMC7871099

[b69] Xu , T. , Sturgeon , D. , Ramirez , J. S. B. , Froudist-Walsh , S. , Margulies , D. S. , Schroeder , C. E. , Fair , D. A. , & Milham , M. P. ( 2019 ). Interindividual variability of functional connectivity in awake and anesthetized rhesus macaque monkeys . Biological Psychiatry. Cognitive Neuroscience and Neuroimaging , 4 ( 6 ), 543 – 553 . 10.1016/j.bpsc.2019.02.005 31072758 PMC7063583

[b70] Xu , T. , Yang , Z. , Jiang , L. , Xing , X.-X. , & Zuo , X.-N. ( 2015 ). A Connectome Computation System for discovery science of brain . Science Bulletin , 60 ( 1 ), 86 – 95 . 10.1007/s11434-014-0698-3

[b71] Yeo , B. T. T. , Krienen , F. M. , Sepulcre , J. , Sabuncu , M. R. , Lashkari , D. , Hollinshead , M. , Roffman , J. L. , Smoller , J. W. , Zöllei , L. , Polimeni , J. R. , Fischl , B. , Liu , H. , & Buckner , R. L. ( 2011 ). The organization of the human cerebral cortex estimated by intrinsic functional connectivity . Journal of Neurophysiology , 106 ( 3 ), 1125 – 1165 . 10.1152/jn.00338.2011 21653723 PMC3174820

[b72] Yushkevich , P. A. , Piven , J. , Hazlett , H. C. , Smith , R. G. , Ho , S. , Gee , J. C. , & Gerig , G. ( 2006 ). User-guided 3D active contour segmentation of anatomical structures: Significantly improved efficiency and reliability . NeuroImage , 31 ( 3 ), 1116 – 1128 . 10.1016/j.neuroimage.2006.01.015 16545965

[b73] Zhang , X. ( 2022 ). Effects of anesthesia on cerebral blood flow and functional connectivity of nonhuman primates . Veterinary Sciences , 9 ( 10 ), 516 . 10.3390/vetsci9100516 36288129 PMC9609818

[b74] Zhao , F. , Pan , H. , Li , N. , Chen , X. , Zhang , H. , Mao , N. , & Ren , Y. ( 2022 ). High-order brain functional network for electroencephalography-based diagnosis of major depressive disorder . Frontiers in Neuroscience , 16 , 976229 . 10.3389/fnins.2022.976229 36017184 PMC9396245

