# Supplementary material for: Systematic cross-species comparison of prefrontal cortex functional networks targeted via transcranial magnetic stimulation
Source: Imaging Neurosci (Camb). 2024 Jul 24;2:imag-2-00243. doi: 10.1162/imag_a_00243 (PMC12272263; doi:10.1162/imag_a_00243)
Supplement: Supplementary Material [file imag_a_00243-supp.pdf]

Supplementary Materials

Additional Metrics for Individual Specific TMS Functional Network Analysis

This study used four metrics to quantify the ability of the TMS-induced electric field to target one of the seven Yeo parcellation networks. The primary metric used within this study is the percentage of overlap between the thresholded binarized functional connectivity maps to the individual functional network parcellations. We calculated overlap as the number of nodes in the maps that overlaid with each functional network divided by the total number of nodes within the functional network. A secondary metric we used to investigate TMS-network stimulation is the DICE Coefficient. The DICE Coefficient quantified the similarity between the binarized thresholded functional connectivity map and each Yeo parcellation. We also calculated the spatial correlation between the simulated map and the Yeo parcellation network. Finally, we looked at the average functional correlation that overlaps with each Yeo parcellation.

Supplemental Figures

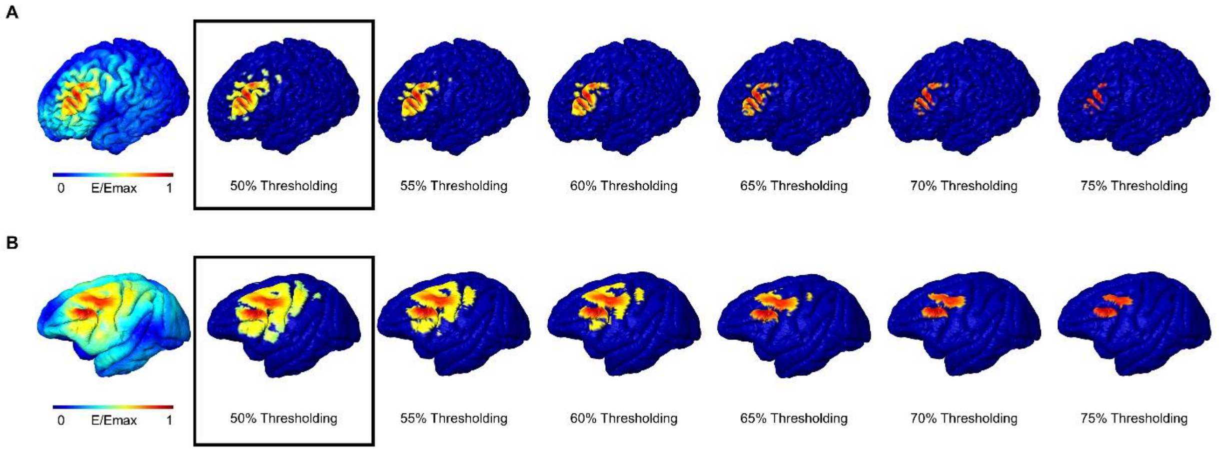

Supplementary Figure 1. Effect of electric field thresholding. A) Electric field distribution for one coil location and orientation on human 32k fsLR cortical surface. A threshold is calculated by identifying the robust maximum of the electric field strength (left) and applying a percentage (50-75%, in 5% increments, shown left to right). All values above this threshold (right) are identified as the seed region for generating the functional connectivity maps. Increased electric field threshold increases the specificity of the seed region. **B)** The electric field distribution on an NHP 32k fsLR cortical surface (left) is shown for one coil configuration (one unique coil location and orientation). The thresholding technique for the NHPs is the same approach outlined for the human models. Like the human models, increasing the electric field thresholding value increases the specificity of the seed region (right)."

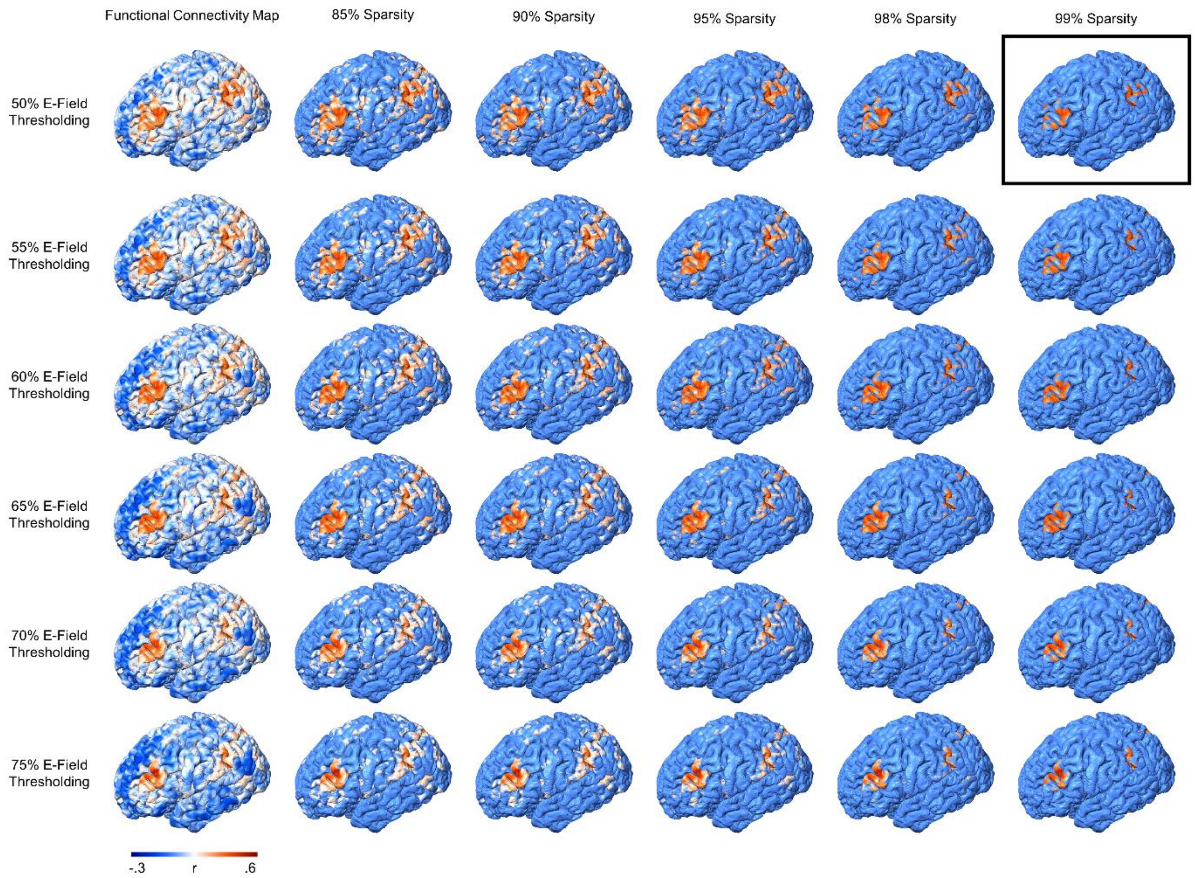

Supplementary Figure 2. Effect of functional connectivity thresholding on a human cortical surface. For higher sparsity thresholds, the functional connectivity seed region becomes more focal. The stimulation hotspots (identified in red) remain spatially the same across all functional and electric field thresholding levels. Increasing the functional connectivity threshold reduces the presence of low-level correlation values in the final TMS-network analysis. The black box indicates the configuration used within this study.

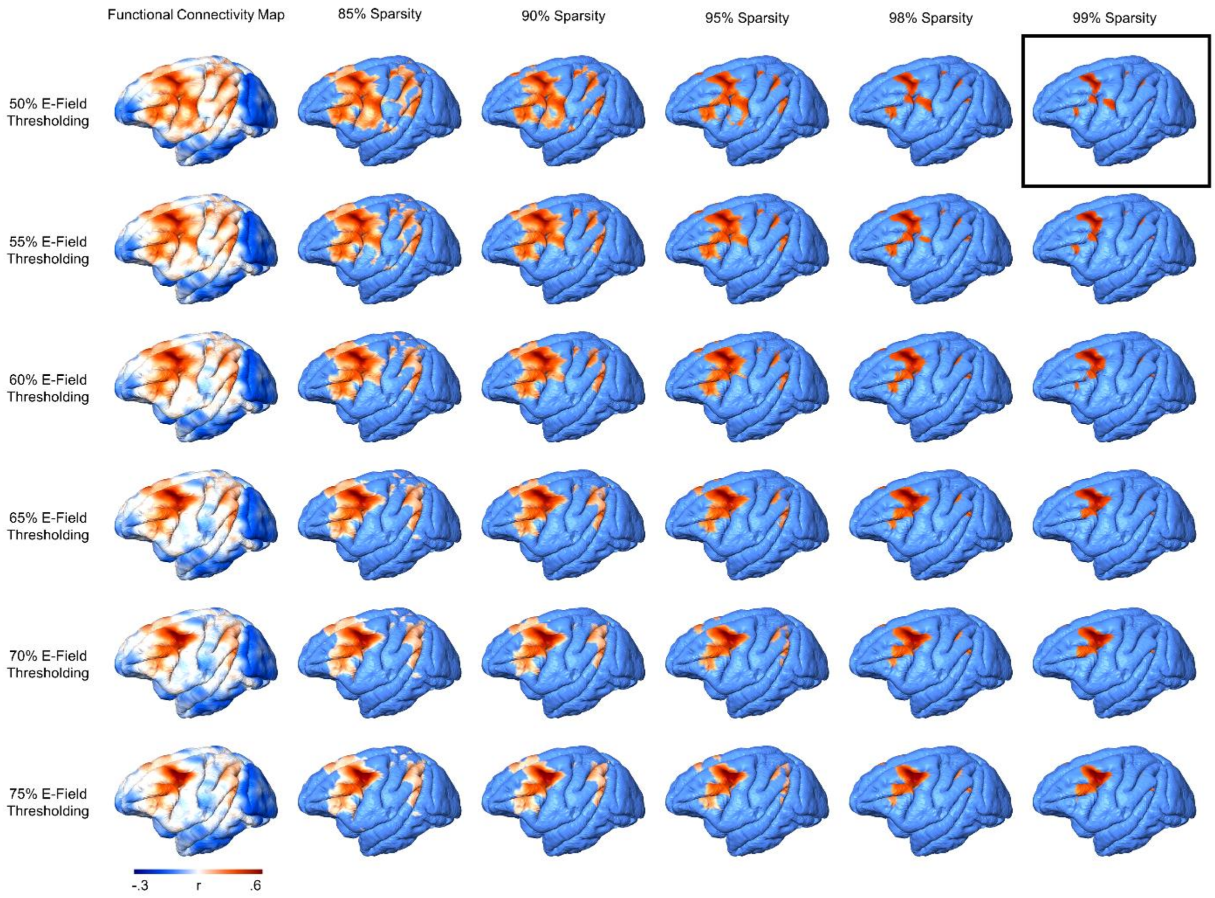

Supplementary Figure 3. Effect of functional connectivity thresholding on a macaque cortical surface. For higher sparsity thresholds, the functional connectivity seed region becomes more focal. For all investigated functional and electric field thresholding levels, the stimulation hotspots (identified in red) remain at the same spatial location. The black box indicates the configuration used within this study.

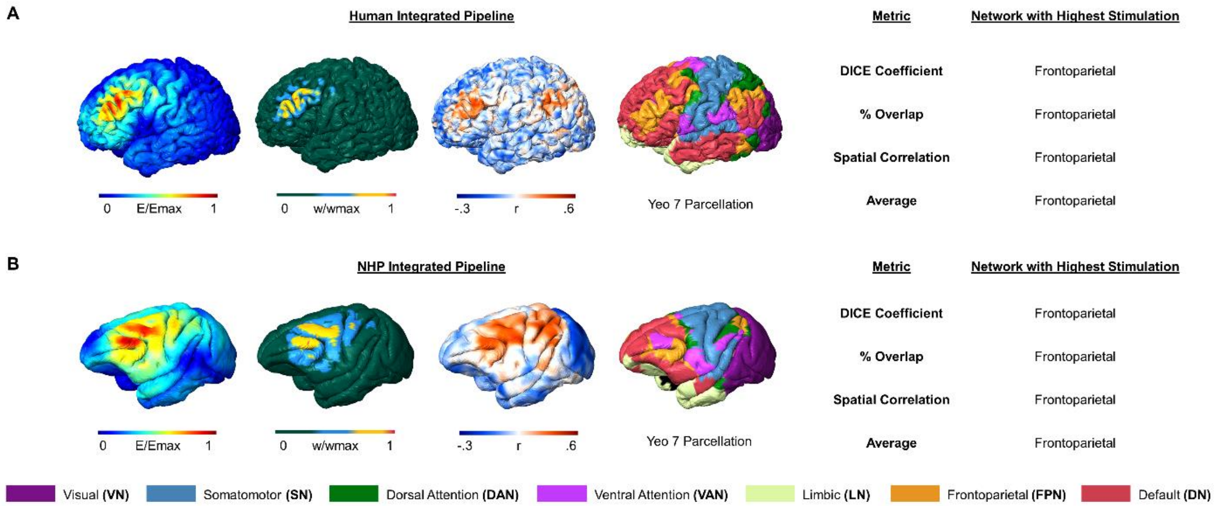

Supplementary Figure 4. Alternative metrics to identify TMS-targeted functional connectivity networks. A) Example human integrated TMS-FC targeting pipeline. Four alternative evaluation metrics were used to identify the FC network with the highest network stimulation (1 – DICE Coefficient, 2 – Percentage of Overlap, 3 – Spatial Correlation, and 4 – Average Stimulation). All methods identified the Frontoparietal Network (FPN) as the network with the highest stimulation for this specific coil location and orientation. **B)** Example NHP integrated TMS-FC targeting pipeline. Four alternative evaluation metrics were used to identify the FC network with the highest network stimulation (1 – DICE Coefficient, 2 – Percentage of Overlap, 3 – Spatial Correlation, and 4 – Average Stimulation). All methods identified the FPN as the network with the highest stimulation for this specific coil location and orientation.

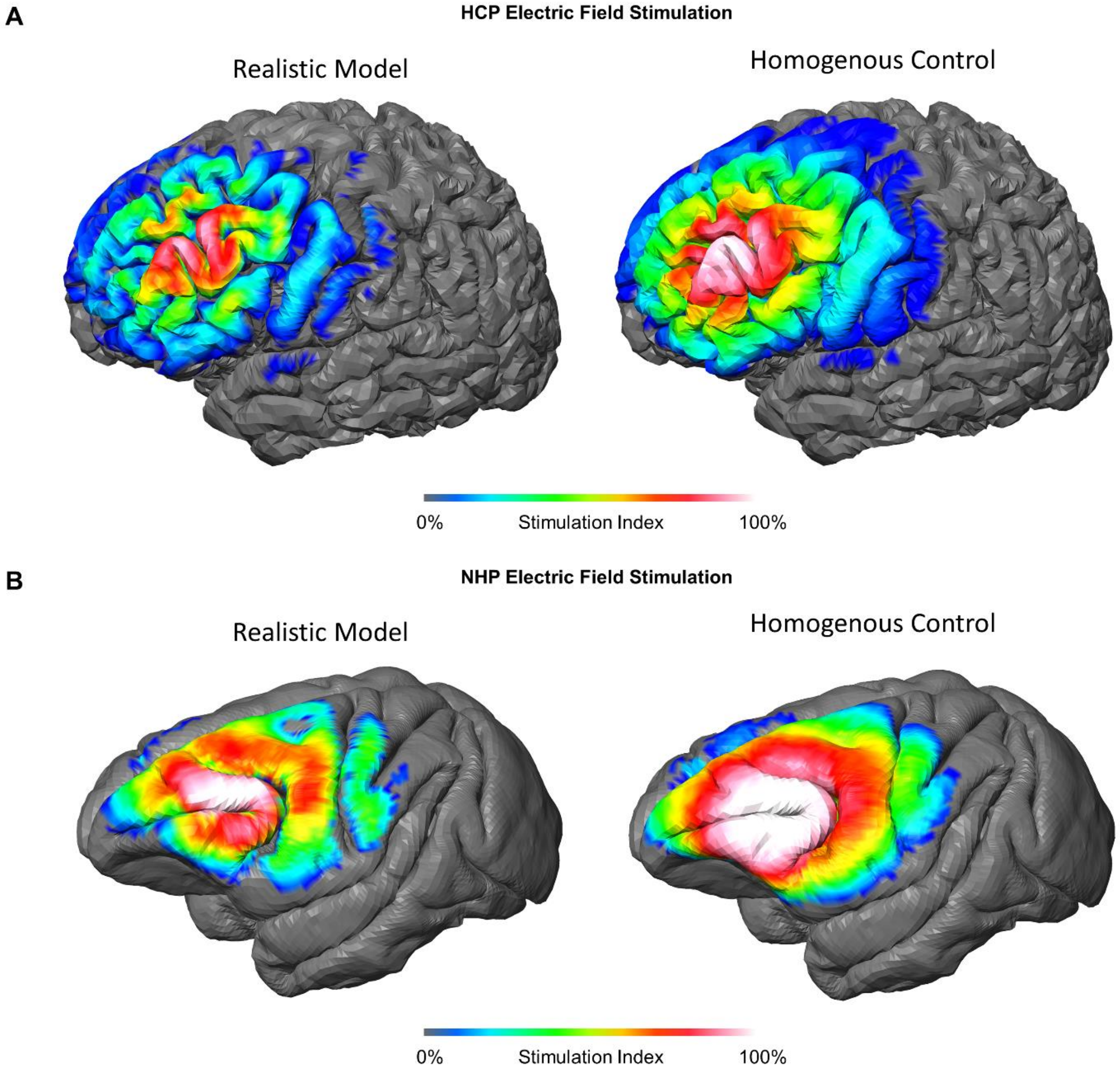

Supplementary Figure 5. Index of stimulation for both realistic and homogenous conductivities. For the realistic conductivity FEM models (left column), the assigned tissue conductivities were $\sigma_{skin} = 0.465 S/m$, $\sigma_{skull} = 0.010 S/m$, $\sigma_{CSF} = 1.654 S/m$, $\sigma_{GM} = 0.276 S/m$, $\sigma_{WM} = 0.126 S/m$. For the homogenous conductivity model (right column), all tissue conductivities were assigned to $\sigma = 0.276 S/m$. **A)** Across all stimulation conditions in the human realistic models, the electric field stimulation index is distributed across the stimulation grid area with the highest area of stimulation at gyral crowns in the center grid region. In the homogenous conductivity models, the stimulation distribution is similar, but with a higher stimulation index under the center of the grid. **B)** Across all simulation conditions in the NHP realistic models, an stimulation preference for the defined prefrontal gryi is identified arising from the CSF-GM interface. In the homogenous conductivity model areas of stimulation are more spread out irrespective of GM anatomy supporting the importance of brain gyrification on targeted brain areas.

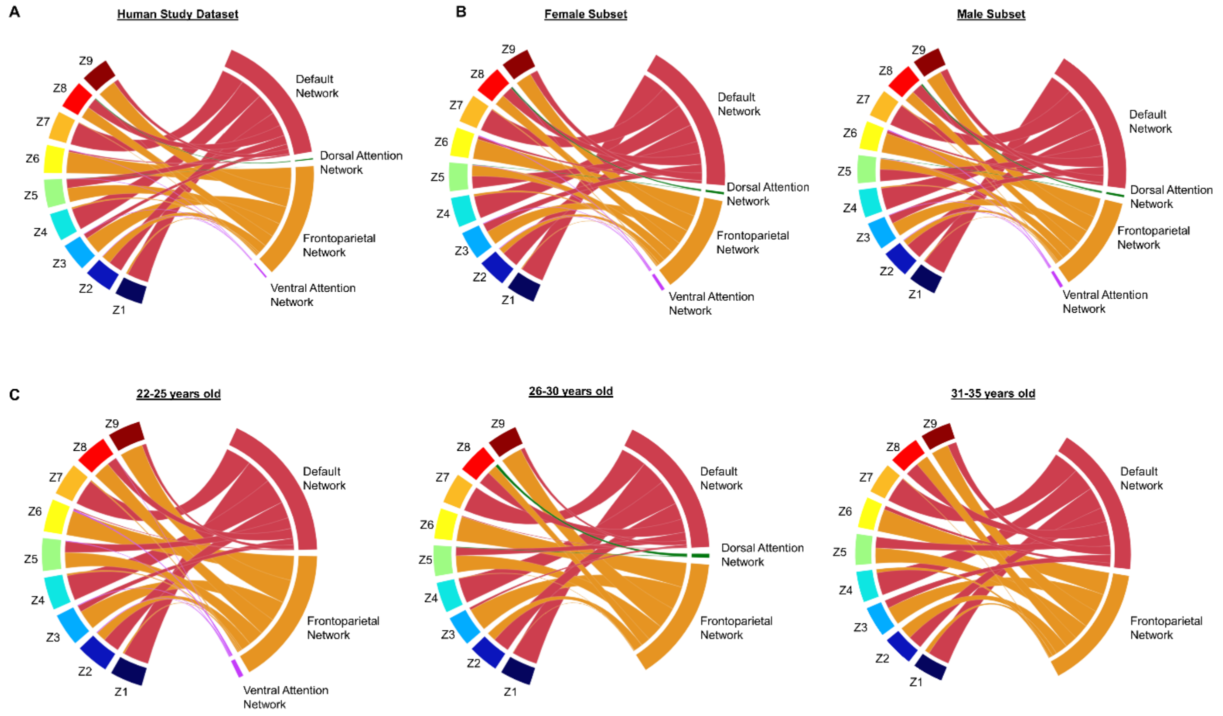

Supplementary Figure 6. Overlap of TMS functional networks with Yeo networks in humans based on demographic subsets. A) Using the entire human dataset, the Frontoparietal Network (FPN) and Default Network (DN) were predominantly targeted. **B)** The human dataset was subdivided by sex (5 male, 5 female) and analyzed. For both subgroups, the FPN and DN were primarily targeted. **C)** The human dataset was subdivided by age (22-25 years old: 4 subjects, 26-30 years old: 3 subjects, 31-35 years old: 3 subjects). When analyzed, each age group reflected a preferential stimulation of the FPN and DN.

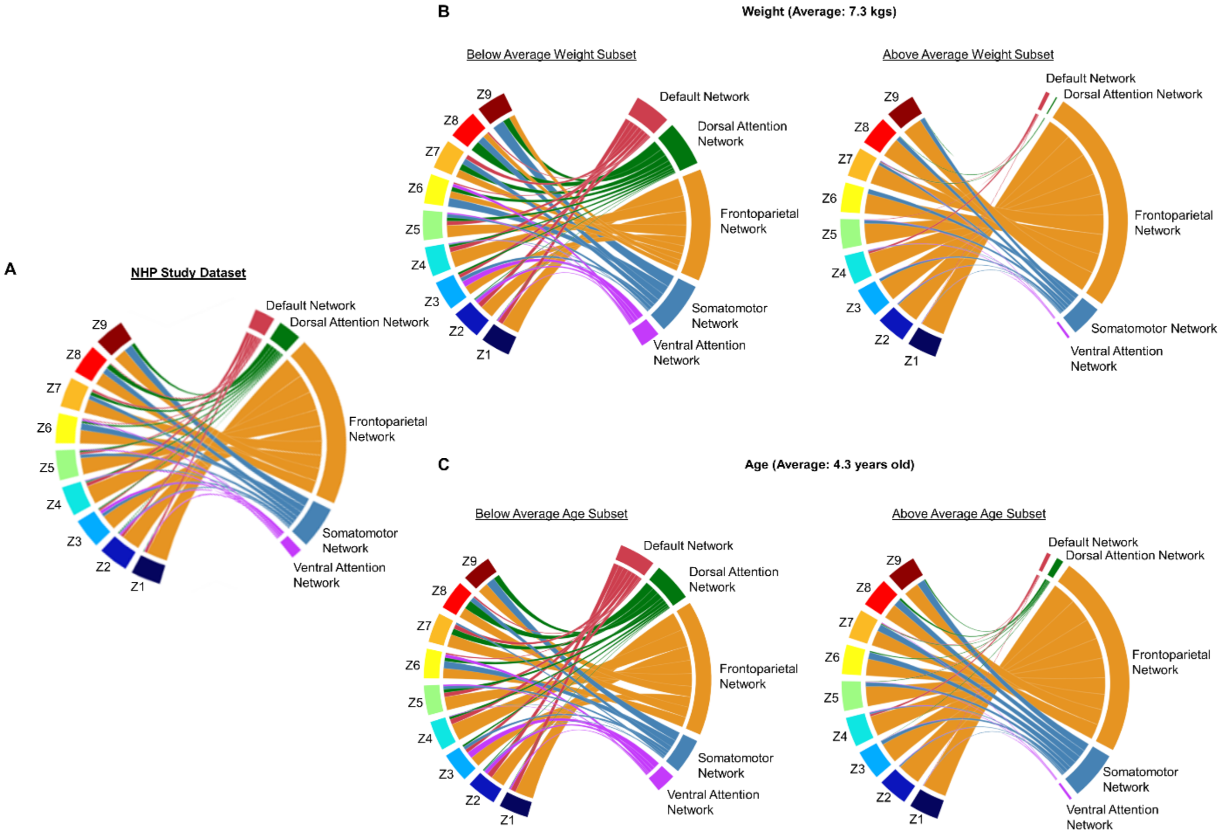

Supplementary Figure 7. Overlap of TMS functional networks with Yeo networks in macaques based on demographic subsets. A) Using the entire macaque dataset, the Frontoparietal Network (FPN) was predominantly stimulated. **B)** The NHP dataset was subdivided by weight (5 above the average weight, 5 below the average weight) and analyzed. For both subgroups, the FPN was primarily stimulated. **C)** The NHP dataset was subdivided by age (5 above the average age, 5 below the average age). When analyzed, each age group reflected a preferential stimulation of the FPN.

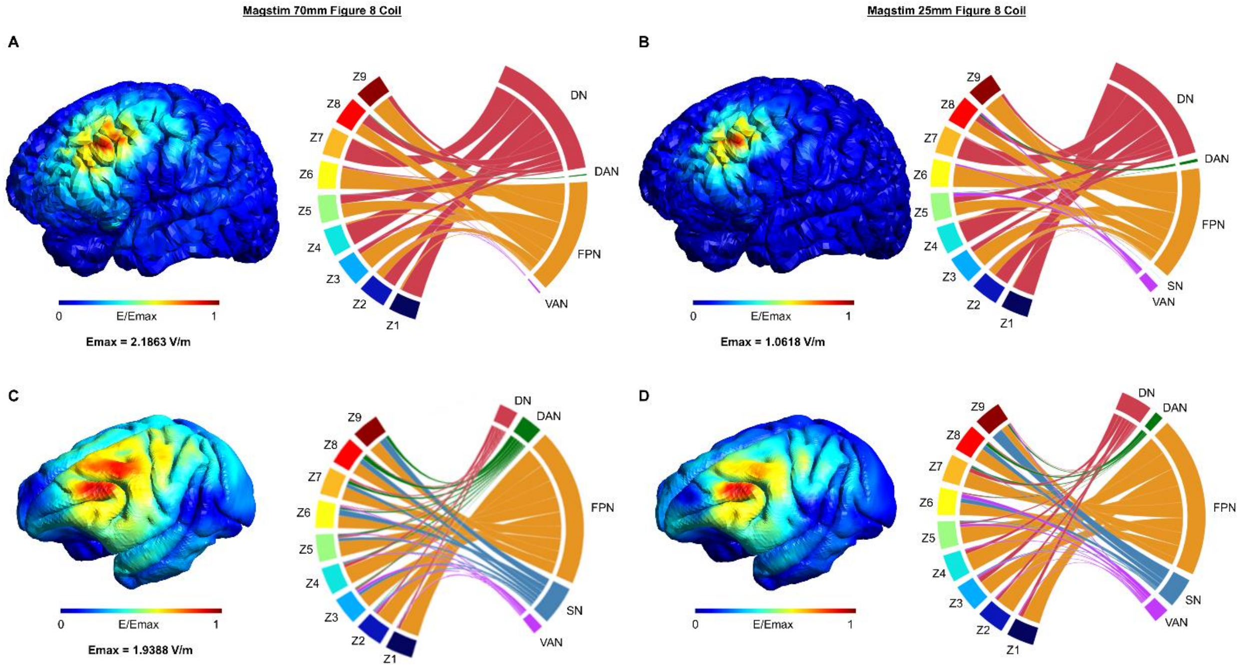

Supplementary Figure 8. Analysis of the effect of TMS coil size on targeted functional networks. A) Using a 70mm Figure-8 coil for the human dataset, the main networks targeted were the Frontoparietal Network (FPN) and the Default Network (DN). **B)** Decreasing the coil size to 25mm resulted in increased electric field focality and decreased electric field intensity. This did not change the targeting preference for the FPN and DN. **C)** Using the 70mm Figure-8 coil on the macaque dataset, the main network targeted was the FPN. **D)** Similarly to the human models, using the a 25mm Figure-8 coil did not result in changes in the main functional network, the FPN.

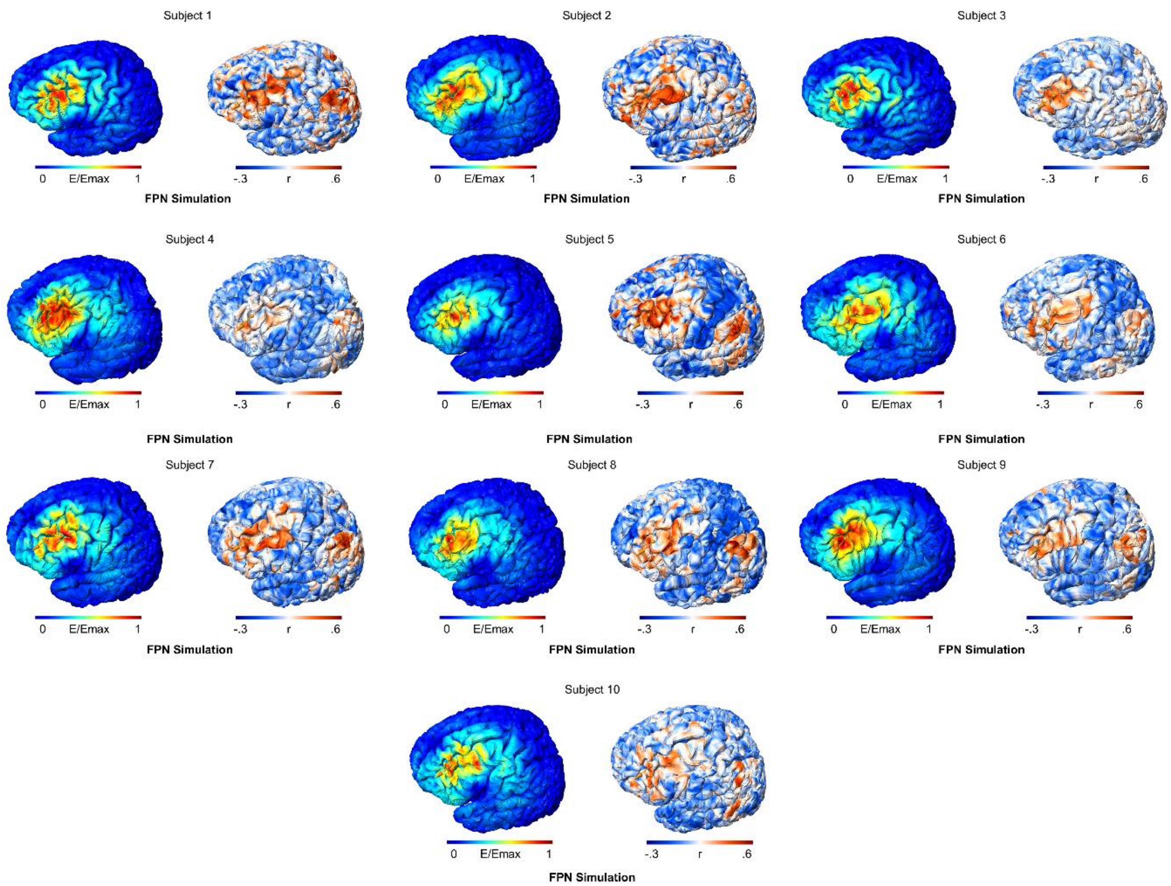

Supplementary Figure 9. TMS-network stimulation results across all subjects for the same coil configuration. Normalized electric field distribution of simulating TMS on the individual FEM volumetric head models at F3, with the coil handle oriented 45° from the midline (right). Each subject's resulting electric field was analyzed to generate subject-specific sparse functional connectivity maps (left). These maps were overlapped with the 7 Yeo networks. For all subjects at this given coil configuration, the network with the highest overlap was the Frontoparietal Network.

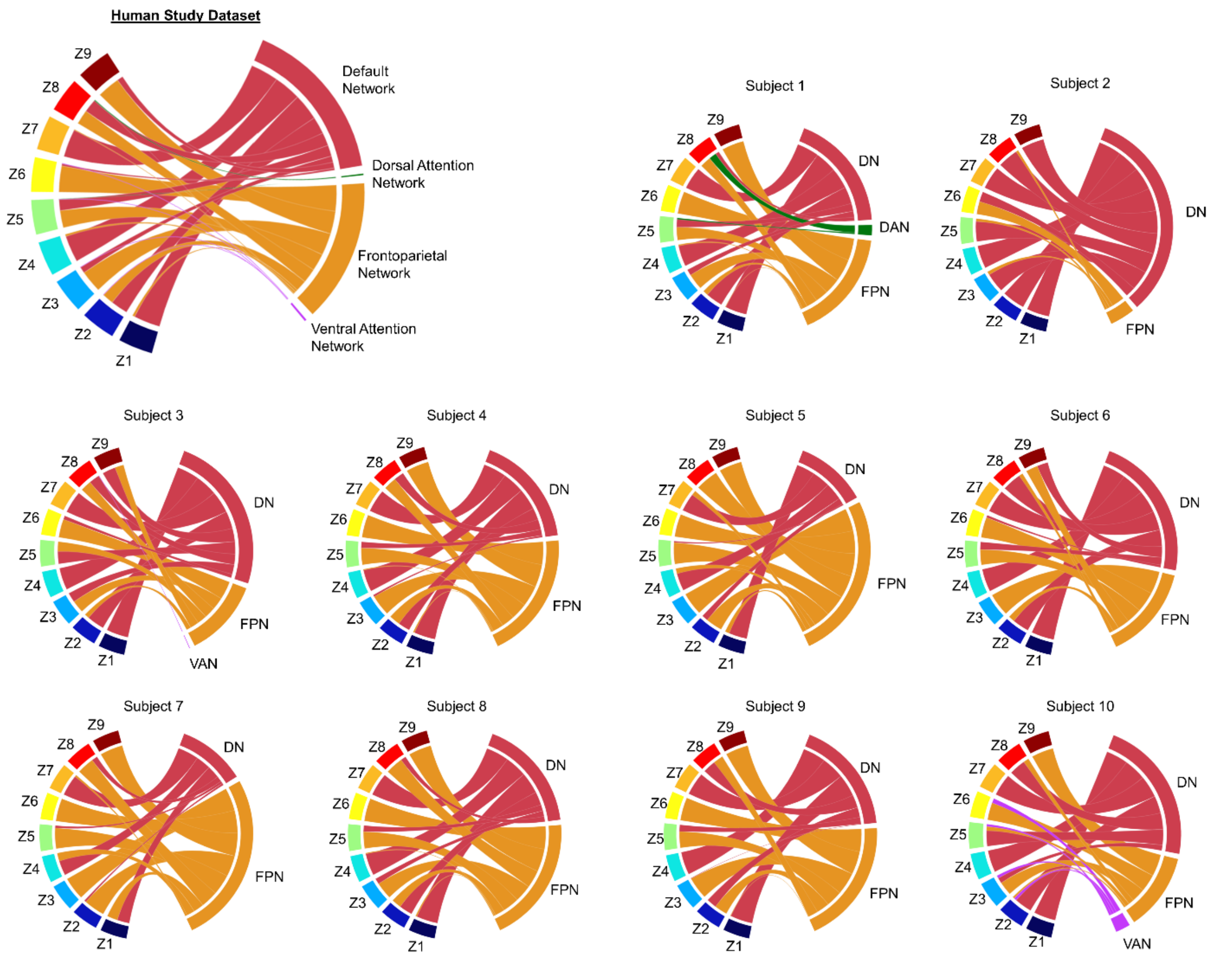

Supplementary Figure 10. TMS-network stimulation results at the individual subject level. Results of an individual subject analysis for each subject within the human dataset compared the group-level analysis results. At the group level, the predominantly targeted networks are the Frontoparietal Network (FPN) and Default Network (DN). In an individual analysis, the FPN and DN networks are still predominantly targeted, however show individual variations in the ratio between DN and FPN.

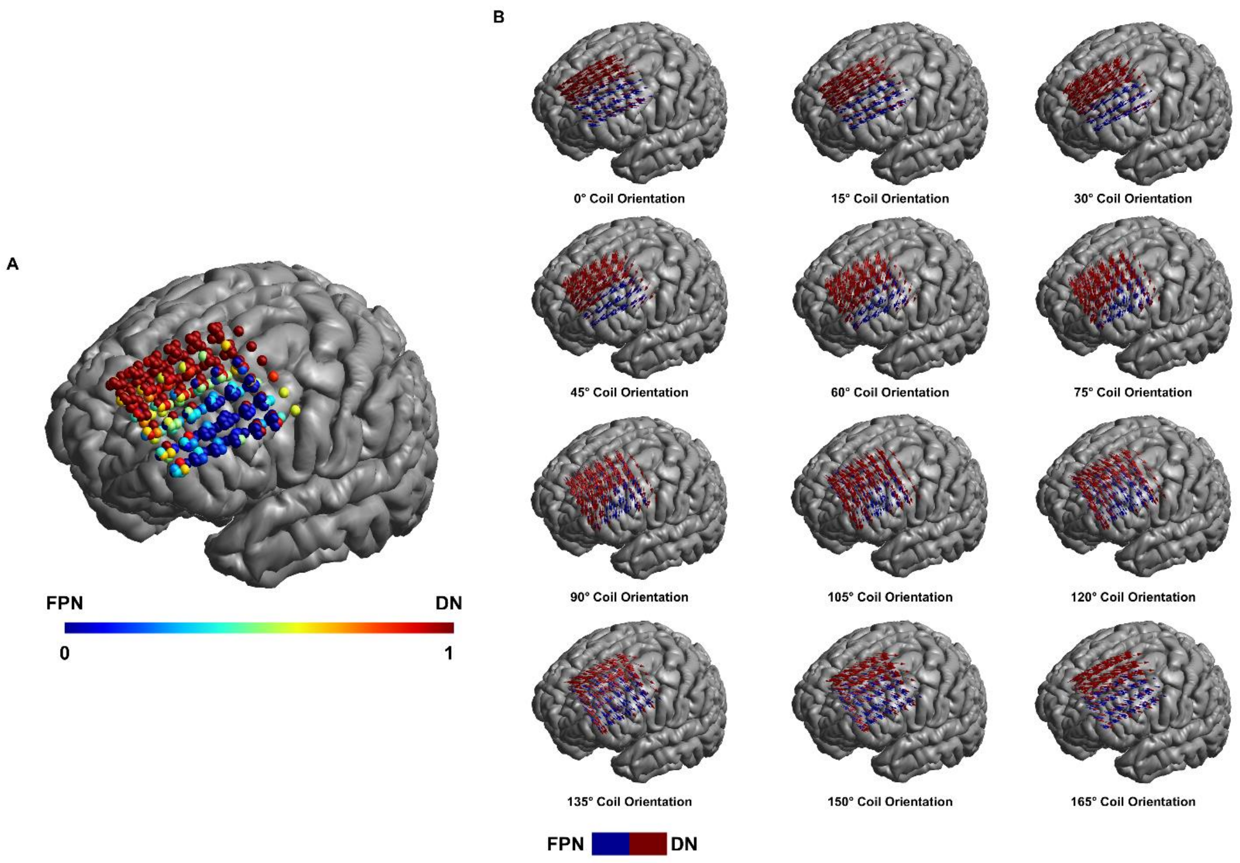

Supplementary Figure 11. TMS-network stimulation results for all subjects and stimulation conditions. Results of all individual subject analyses for each subject within the human dataset. To analyze coil locations, we analyzed the percentage of orientations per position that fell within the Default Network (DN) or Frontoparietal Network (FPN) (100% DN relates to 1 [red], 100% FPN relates to 0 [blue]). To analyze coil orientations, we visualized the coil orientations per position that were stimulating the DN (red) and FPN (blue) for each coil orientation.
